# Supplementary material for: Impact of yoga on cardiometabolic health in adults with overweight or obesity: A systematic review and meta-analysis of randomized controlled trials
Source: PLOS Glob Public Health. 2026 Apr 22;6(4):e0006174. doi: 10.1371/journal.pgph.0006174 (PMC13102220; doi:10.1371/journal.pgph.0006174)
Supplement: S1 Text — (PDF) [file pgph.0006174.s001.pdf]

Medline, PsycINFO, EMBASE (OVID)
1.
Yoga.sh.
2.
yogic.tw.
3.
yoga.tw.
4.
asana.tw.
5.
pranayam*.tw.
6.
dhyan*.tw.
7.
shavasan*.tw.
8.
meditation.tw.
9.
mantra.tw.
10.
1 or 2 or 3 or 4 or 5 or 6 or 7 or 8 or 9
11.
blood pressure.xm.
12.
prehypertension.sh.
13.
arterial pressure/
14.
systol*.tw.
15.
diastol*.tw.
16.
bp response.tw.
17.
bp change.tw.
18.
bp increase.tw.
19.
bp decrease.tw.
20.
bp reduction.tw.
21.
bp monitor*.tw.
22.
bp measurement*.tw.
23.
bp improv*.tw.
24.

hypertension.xm.
25.
hypertensi*.tw.
26.
prehypertensi*.tw.
27.
11 or 12 or 13 or 14 or 15 or 16 or 17 or 18 or 19 or 20 or 21 or 22 or 23 or 24 or 25 or 26
28.
((plasma or blood) and glucose).mp. or blood glucose/ or (FBG or PPG or PBG or PPBG or glycaem* or glycat*).mp.
29.
glucose metabolism disorders.xm.
30.
hba1c.tw.
31.
28 or 29 or 30
32.
lipoproteins.xm.
33.
cholesterol.xm.
34.
triglycerides.sh.
35.
Lipid Metabolism Disorders.xm.
36.
HDL.tw.
37.
LDL.tw.
38.
TG.tw.
39.
VLDL.tw.
40.
triacylglycer*.tw.
41.
TC.tw.
42.
cholesterol*.tw.
43.
lipid*.tw.
44.
lipoprotein*.tw.
45.
Triglycerid*.tw.
46.
hypertriglyceridemia*.tw.
47.
Hypercholesterolemia*.tw.

48.
Hyperlipidemia*.tw.
49.
low-density lipoprotein*.tw.
50.
high-density lipoprotein*.tw.
51.
very-low-density lipoprotein*.tw.
52.
low density lipoprotein*.tw.
53.
high density lipoprotein*.tw.
54.
very-low-density lipoprotein*.tw.
55.
TAG.tw.
56.
32 or 33 or 34 or 35 or 36 or 37 or 38 or 39 or 40 or 41 or 42 or 43 or 44 or 45 or 46 or 47 or 48 or 49 or 50 or 51 or 52 or 53 or 54 or 55
57.
exp Inflammation/
58.
c-reactive protein.mp. or exp C-Reactive Protein/
59.
tumor necrosis factor.mp. or exp Tumor Necrosis Factor-alpha/
60.
exp Interleukin-6/
61.
interleukin.mp. or exp Interleukins/
62.
IL-6.mp.
63.
IL-1.mp. or exp Interleukin-1/
64.
IL-10.mp. or exp Interleukin-10/
65.
N-terminal pro-brain natriuretic peptide.mp.
66.
NT-ProBNP.mp.
67.
exp Leptin/ or leptin.mp.
68.
resistin.mp. or Resistin/
69.
omentin.mp. or exp Adipokines/
70.
Thiobarbituric acid-reactive substances.mp. or exp Thiobarbituric Acid Reactive Substances/
71.

malondialdehyde.mp. or exp Malondialdehyde/
72.
MDA.mp.
73.
F2-isoprostanes.mp. or exp F2-Isoprostanes/
74.
F2-isoPs.mp.
75.
oxidative stress.mp. or exp Oxidative Stress/
76.
Lipid peroxidation.mp. or exp Lipid Peroxidation/
77.
LIPOX.mp.
78.
antioxidant.mp. or exp Antioxidants/
79.
3-Nitrotyrosine.mp.
80.
3-NT.mp.
81.
Hydrogen peroxide.mp. or exp Hydrogen Peroxide/
82.
exp Sulfhydryl Compounds/ or Sulfhydryl.mp.
83.
Myeloperoxidase.mp. or exp Peroxidase/
84.
MPO.mp. or exp Peroxidases/
85.
POVPC.mp.
86.
PGPC.mp.
87.
Superoxide dismutase.mp. or exp Superoxide Dismutase/ or SOD.mp.
88.
total antioxidant capacity.mp.
89.
TAC.mp.
90.
trolox equivalent antioxidant capacity.mp.
91.
TEAC.mp.
92.
Glutathione peroxidase.mp. or exp Glutathione Peroxidase/
93.
Glutathione/ or Glutathione.mp.
94.
Catalase.mp. or exp Catalase/
95.

ascorbic acid.mp. or exp Ascorbic Acid/
96.
Nitric oxide.mp. or exp Nitric Oxide/
97.
Adenosine deaminase.mp. or exp Adenosine Deaminase/
98.
57 or 58 or 59 or 60 or 61 or 62 or 63 or 64 or 65 or 66 or 67 or 68 or 69 or 70 or 71 or 72 or 73 or 74 or 75 or 76 or 77 or 78 or 79 or 80 or 81 or 82 or 83 or 84 or 85 or 86 or 88 or 89 or 90 or 91 or 92 or 93 or 94 or 95 or 96 or 97
99.
27 or 31 or 56 or 98
100.
10 and 99

Scopus
( ( TITLE-ABS-KEY ( ( ( ( blood OR arterial ) AND pressure ) OR prehypertension OR systol* OR diastol* OR ( bp AND ( response OR change OR increase OR decrease OR reduction OR monitor* OR measurement* OR improv* ) ) OR hypertension OR hypertensi* OR prehypertensi* ) ) ) OR ( TITLE-ABS-KEY ( ( ( ( plasma OR blood ) AND glucose ) OR fbg OR ppg OR pbg OR ppbg OR glycaem* OR glycat* OR ( glucose AND metabolism ) ) ) ) OR ( TITLE-ABS-KEY ( ( lipoproteins OR cholesterol OR triglycerides OR ( lipid AND metabolism AND disorder* ) OR hdl OR ldl OR tg OR vldl OR triacylglycer* OR tc OR cholesterol* OR lipid* OR lipoprotein* OR triglycerid* OR hypertriglyceridemia* OR hypercholesterolemia* OR hyperlipidemia* OR low-density AND lipoprotein* OR high-density AND lipoprotein* OR very-low-density AND lipoprotein* OR low AND density AND lipoprotein* OR high AND density AND lipoprotein* OR very-low-density AND lipoprotein* OR tag ) ) ) OR ( TITLE-ABS-KEY ( ( inflammation OR ( c-reactive AND protein ) OR ( tumor AND necrosis AND factor ) OR interleukin* OR il-1 OR il-6 OR il-10 OR ( n-terminal AND pro-brain AND natriuretic AND peptide ) OR nt-probnp OR leptin OR resistin OR omentin OR adipokines OR ( thiobarbituric AND acid-reactive AND substanc* ) OR tbars OR malondialdehyde OR mda OR f2-isoprostanes OR f2-isops OR ( oxidative AND stress ) OR ( lipid AND peroxidation ) OR lipox OR 3-nitrotyrosine OR 3-nt OR ( hydrogen AND peroxide ) OR sulfhydryl OR myeloperoxidase OR peroxidase* OR mpo OR povpc OR pgpc OR antioxidant OR ( superoxide AND dismutase ) OR sod OR ( total AND antioxidant AND capacity ) OR tac OR ( trolox AND equivalent AND antioxidant AND capacity ) OR teac OR ( glutathione AND peroxidase ) OR glutathione OR catalase OR ( ascorbic AND acid ) OR ( nitric AND oxide ) OR ( adenosine AND deaminase ) ) ) ) ) ) AND ( TITLE-ABS-KEY ( ( yogic OR yoga OR asana OR pranayam* OR dhyan* OR shavasan* OR meditation OR mantra ) ) )

SportDISCUS (EBSCOhost)
S1
TX ((Yoga OR yogic OR yoga OR asana OR pranayam* OR dhyan* OR shavasan* OR meditation OR mantra))
S2
TX ((((blood OR arterial) AND pressure) OR prehypertension OR systol* OR diastol* OR (bp AND (response OR change OR increase OR decrease OR reduction OR monitor* OR measurement* OR improv*)) OR hypertension OR hypertensi* OR prehypertensi*))
S3
TX ((((plasma or blood) and glucose) or FBG or PPG or PBG or PPBG or glycaem* or glycat* OR (glucose AND metabolism)))
S4
TX (lipoproteins OR cholesterol OR triglycerides OR (Lipid AND Metabolism) OR HDL OR LDL OR TG OR VLDL OR triacylglycer* OR TC OR cholesterol* OR lipid* OR lipoprotein* OR Triglycerid* OR hypertriglyceridemia* OR Hypercholesterolemia* OR Hyperlipidemia* OR ((low OR high) AND density AND Lipoprotein*) OR TAG)
S5
TX ((Inflammation OR (c-reactive AND protein) OR (tumor AND necrosis AND factor) OR interleukin* OR IL-1 OR IL-6 OR IL-10 OR (N-terminal AND pro-brain AND natriuretic AND peptide) OR NT-ProBNP OR Leptin OR resistin OR omentin OR Adipokines OR (Thiobarbituric AND acid-reactive AND substanc*) OR TBARS OR malondialdehyde OR MDA OR F2-isoprostanes OR F2-isoPs OR (oxidative AND stress) OR (Lipid AND peroxidation) OR LIPOX OR 3-Nitrotyrosine OR 3-NT OR (Hydrogen AND peroxide) OR Sulfhydryl OR Myeloperoxidase OR Peroxidase* OR MPO OR POVPC OR PGPC OR antioxidant OR (Superoxide AND dismutase) OR SOD OR (total AND antioxidant AND capacity) OR TAC OR (trolox AND equivalent AND antioxidant AND capacity) OR TEAC OR (Glutathione AND peroxidase) OR Glutathione OR Catalase OR (ascorbic AND acid) OR (Nitric AND oxide) OR (Adenosine AND deaminase))
S6
S2 OR S3 OR S4 OR S5
S7
S1 AND S6
