# Supplementary material for: Impact of yoga on cardiometabolic health in adults with overweight or obesity: A systematic review and meta-analysis of randomized controlled trials
Source: PLOS Glob Public Health. 2026 Apr 22;6(4):e0006174. doi: 10.1371/journal.pgph.0006174 (PMC13102220; doi:10.1371/journal.pgph.0006174)
Supplement: S1 Table — (DOCX) [file pgph.0006174.s003.docx]

|  | Database Searches  Study | Title | Reason |
| --- | --- | --- | --- |
| 1 | Djelic 2011 | Positive impact of yoga exercise program for female seniors on risk profiles of cardiovascular diseases | Report not retrieved |
| 2 | Sharma 2015 | Poster Session IIIWednesday, December 9, 2015 | Report not retrieved |
| 3 | Smith 2012 | Poster Session III | Report not retrieved |
| 4 | Tolahunase 2018 | Impact of yoga- and meditation-based lifestyle intervention on depression and quality of life in infertile couples: a randomized controlled trial | Report not retrieved |
| 5 | Bhaskar 2023 | Effects of Sudarshan KriyaYoga and Advanced Meditation Program on Genetic Expression of Pro-inflammatory and Antioxidants Genes | Wrong comparator |
| 6 | Balakrishnan 2022 | A RANDOMIZED CASE-CONTROL PILOT STUDY ON THE NEUROCHEMICAL BASIS OF PAIN MODULATION IN PATIENTS WITH MIGRAINE, WHO PRACTICED INTEGRATED AMRITA MEDITATION TECHNIQUE | Wrong intervention |
| 7 | Bhargava 1988 | Autonomic responses to breath holding and its variations following pranayama | Wrong intervention |
| 8 | Griebeler 2022 | The use of virtual visits for obesity pharmacotherapy in patients with overweight or obesity compared with in-person encounters | Wrong intervention |
| 9 | R 2023 | An interprofessional collaborative approach to alleviate workplace stress among medical college faculty during COVID pandemic | Wrong intervention |
| 10 | Raja-Khan 2017 | Mindfulness-Based Stress Reduction in Women with Overweight or Obesity: A Randomized Clinical Trial | Wrong intervention |
| 11 | RodriguesdeOliveira 2021 | Mindfulness meditation training effects on quality of life, immune function and glutathione metabolism in service healthy female teachers: A randomized pilot clinical trial | Wrong intervention |
| 12 | Saban 2022 | Impact of a Mindfulness-Based Stress Reduction Program on Psychological Well-Being, Cortisol, and Inflammation in Women Veterans | Wrong intervention |
| 13 | Schneider 2021 | Randomized controlled trial of stress reduction with meditation and health education in black men and women with high normal and normal blood pressure | Wrong intervention |
| 14 | Sharma 2013 | Early interventions for diabetes and dysglycaemia. Surgery in the treatment of obesity and diabetes. Abstracts of the 5th International Congress on Prediabetes and the Metabolic Syndrome. Vienna, Austria. April 18-20, 2013 | Wrong intervention |
| 15 | Subramanian 2022 | Effect of 4-Week Heartfulness Meditation on Stress Scores, Sleep Quality, and Oxidative and Inflammatory Biochemical Parameters in COVID-19 Patients after Completion of Standard Treatment - A Randomized Controlled Trial | Wrong intervention |
| 16 | Turankar 2013 | Effects of slow breathing exercise on cardiovascular functions, pulmonary functions & galvanic skin resistance in healthy human volunteers - a pilot study | Wrong intervention |
| 17 | Akshaya 2018 | Effect of Bhastrika Pranayama on peak flow rate in wheezing patients | Wrong outcomes |
| 18 | Black 2013 | Yogic meditation reverses NF-Œ∫B and IRF-related transcriptome dynamics in leukocytes of family dementia caregivers in a randomized controlled trial | Wrong outcomes |
| 19 | Bussaraporn 2022 | Comparative Effectiveness of Ruesi Dadton (Thai Yoga) Exercise and Stretching Exercise in Office Workers | Wrong outcomes |
| 20 | Agnihotri 2013 | Significance of yoga in asthma management to improve the status of quality of life | Wrong patient population |
| 21 | Ajjimaporn 2018 | Effects of 8 weeks of modified hatha yoga training on resting-state brain activity and the p300 ERP in patients with physical disability-related stress | Wrong patient population |
| 22 | Akhani 2019 | A study of cardiorespiratory efficiency following yoga in healthy Indian medical students | Wrong patient population |
| 23 | Bal 2015 | EFFECTS OF SHORT TERM PRACTICE OF BHASTRIKA PRANAYAMA ON METABOLIC FITNESS (METF) AND BONE INTEGRITY (BI) | Wrong patient population |
| 24 | Balakrishnan 2011 | EFFECT OF COMBINED ACTION OF YOGASANAS AND PRANAYAMA EXERCISES REDUCES LIPID PROFILES AND ENHANCES ANTIOXIDANT STATUS IN YOUNG HEALTHY INDIVIDUALS | Wrong patient population |
| 25 | Bargal 2022 | Evaluation of the Effect of Left Nostril Breathing on Cardiorespiratory Parameters and Reaction Time in Young Healthy Individuals | Wrong patient population |
| 26 | Bernardi 2001 | Effect of rosary prayer and yoga mantras on autonomic cardiovascular rhythms: comparative study | Wrong patient population |
| 27 | Bharshankar 2003 | Effect of yoga on cardiovascular system in subjects above 40 years | Wrong patient population |
| 28 | Bhatnagar 2015 | Physiological Responses of Yogic Breathing Exercise in Young Females | Wrong patient population |
| 29 | Bhattacharya 2002 | Improvement in oxidative status with yogic breathing in young healthy males | Wrong patient population |
| 30 | Bhavanani 2004 | Effect of six weeks of shavasan training on spectral measures of short-term heart rate variability in young healthy volunteers | Wrong patient population |
| 31 | Bidwell 2012 | Yoga training improves quality of life in women with asthma | Wrong patient population |
| 32 | Birdee 2015 | Yoga Combined With Health Education for Risk Reduction of Metabolic Syndrome: A Randomized Controlled Pilot Feasibility Study | Wrong patient population |
| 33 | Biswas 2015 | Antistress Activity of Tinospora Cordifolia with Application of Yoga | Wrong patient population |
| 34 | Blumenthal 1989 | Cardiovascular and behavioral effects of aerobic exercise training in healthy older men and women | Wrong patient population |
| 35 | Boroujeni 2015 | Yoga intervention on blood NO in female migraineurs | Wrong patient population |
| 36 | Carranque 2012 | Hematological and biochemical modulation in regular yoga practitioners | Wrong patient population |
| 37 | Chang 2014 | The effect of short term yoga and Tai-Chi education exercise on antioxidant capacity and oxidative stress measures | Wrong patient population |
| 38 | Chanta 2022 | Effect of Hatha yoga training on rhinitis symptoms and cytokines in allergic rhinitis patients | Wrong patient population |
| 39 | Chen 2016 | Effects of 8-Week Hatha Yoga Training on Metabolic and Inflammatory Markers in Healthy, Female Chinese Subjects: a Randomized Clinical Trial | Wrong patient population |
| 40 | Cheung 2018 | Effects of yoga on oxidative stress, motor function, and non-motor symptoms in Parkinson's disease: a pilot randomized controlled trial | Wrong patient population |
| 41 | Chhugani 2018 | Effects of Integrated Yoga Intervention on Psychopathologies and Sleep Quality Among Professional Caregivers of Older Adults With Alzheimer's Disease: A Controlled Pilot Study | Wrong patient population |
| 42 | Ciosek 2015 | Is training yoga regulary can have an effective impact on dealing with stress? | Wrong patient population |
| 43 | Cohen 2011 | Iyengar yoga versus enhanced usual care on blood pressure in patients with prehypertension to stage I hypertension: a randomized controlled trial | Wrong patient population |
| 44 | Cohen 2016 | Blood Pressure Effects of Yoga, Alone or in Combination With Lifestyle Measures: results of the Lifestyle Modification and Blood Pressure Study (LIMBS) | Wrong patient population |
| 45 | CotaESouza 2023 | Yoga practice can reduce metabolic syndrome and cardiovascular risk in climacteric women | Wrong patient population |
| 46 | Gawrys 2020 | Yoga practitioners body composition and health related indicators | Wrong patient population |
| 47 | Granath 2006 | Stress management: a randomized study of cognitive behavioural therapy and yoga | Wrong patient population |
| 48 | Harinath 2004 | Effects of Hatha yoga and Omkar meditation on cardiorespiratory performance, psychologic profile, and melatonin secretion | Wrong patient population |
| 49 | Jones 2016 | A yoga & exercise randomized controlled trial for vasomotor symptoms: Effects on heart rate variability | Wrong patient population |
| 50 | Kanaya 2014 | Restorative yoga and metabolic risk factors: the Practicing Restorative Yoga vs. Stretching for the Metabolic Syndrome (PRYSMS) randomized trial | Wrong patient population |
| 51 | Kanojia 2013 | Effect of yoga on autonomic functions and psychological status during both phases of menstrual cycle in young healthy females | Wrong patient population |
| 52 | Kim 2014 | Effects of yogic exercises on life stress and blood glucose levels in nursing students | Wrong patient population |
| 53 | Kothari 2023 | Exploring the Effect of Yoga on Exercise Endurance As Assessed by Cardiorespiratory Efficiency Tests in Exercise Physiology Laboratory: A Pilot Study | Wrong patient population |
| 54 | Kumar 2021 | Study on yoga practices and biochemical, physical and physiological alterations: A perspective on yoga as preventive strategy against COVID 19 | Wrong patient population |
| 55 | Kunal 2018 | Impact of regularly supervised training of pranayama and omkar meditation on the cardio-respiratory parameters and short-term memory of persons with special needs | Wrong patient population |
| 56 | Kuppusamy 2016 | Immediate effects of Bhramari pranayama on resting cardiovascular parameters in healthy adolescents | Wrong patient population |
| 57 | Lai 2023 | The Effects of Yoga Exercise on Blood Pressure and Hand Grip Strength in Chronic Stroke Patients: A Pilot Controlled Study | Wrong patient population |
| 58 | Lim 2015 | Regular Yoga Practice Improves Antioxidant Status, Immune Function, and Stress Hormone Releases in Young Healthy People: A Randomized, Double-Blind, Controlled Pilot Study | Wrong patient population |
| 59 | Manna 2018 | Effects of Yoga Training on Body Composition and Oxidant-Antioxidant Status among Healthy Male | Wrong patient population |
| 60 | McCaffrey 2005 | The effects of yoga on hypertensive persons in Thailand | Wrong patient population |
| 61 | Misra 2019 | Take a deep breath: a randomized control trial of Pranayama breathing on uncontrolled hypertension | Wrong patient population |
| 62 | Nagarathna 2021 | Effectiveness of Yoga Lifestyle on Lipid Metabolism in a Vulnerable Population-A Community Based Multicenter Randomized Controlled Trial | Wrong patient population |
| 63 | Packyanathan 2020 | Comparison of the effect of Yoga, Zumba and Aerobics in controlling blood pressure in the Indian population | Wrong patient population |
| 64 | Papp 2016 | Effects of High-Intensity Hatha Yoga on Cardiovascular Fitness, Adipocytokines, and Apolipoproteins in Healthy Students: A Randomized Controlled Study | Wrong patient population |
| 65 | Parthiban 2020 | DECREASING FASTING BLOOD SUGAR LEVEL THROUGH YOGIC PRACTICES AND PHYSICAL EXERCISES: a 8 WEEKS PROSPECTIVE STUDY FOR THE MIDDLE AGED MEN | Wrong patient population |
| 66 | Qian 2023 | REFLECTIONS OF YOGA PRACTICE ON THE PHYSICAL PERFORMANCE OF OBESE STUDENTS | Wrong patient population |
| 67 | Raghuraj 2008 | Immediate effect of specific nostril manipulating yoga breathing practices on autonomic and respiratory variables | Wrong patient population |
| 68 | Rajbhoj 2015 | Effect of yoga module on pro-inflammatory and anti-inflammatory cytokines in industrial workers of lonavla: a randomized controlled trial | Wrong patient population |
| 69 | Rajbhoj 2023 | The Effects of Yoga Practice on Lung Function and sIL-2R Biomarkers in Individuals Working and Living in the Lonavala Industrial Area: A Randomized Controlled Trial | Wrong patient population |
| 70 | Rankhambe 2021 | Effect of Sudarshan Kriya Yoga on cold pressor response in healthy young adults | Wrong patient population |
| 71 | Rao 2023 | Efficacy Of Yogic Practices On Systolic And Diastolic Blood Pressure Among Migraine Sufferers | Wrong patient population |
| 72 | Ray 2001 | Effect of yogic exercises on physical and mental health of young fellowship course trainees | Wrong patient population |
| 73 | Ray 2023 | Session of yoga, with and without slow (Ujjayi) breathing, reduces anxiety; no change on acute pain sensitivity and endogenous pain modulation | Wrong patient population |
| 74 | Riley 2017 | Improving physical and mental health in frontline mental health care providers: Yoga-based stress management versus cognitive behavioral stress management | Wrong patient population |
| 75 | Rohini 2022 | Immediate effects of the practise of Sheethali pranayama on heart rate and blood pressure parameters in healthy volunteers | Wrong patient population |
| 76 | S 2017 | Comparative study on the effect of yogic relaxing asanas and pranayamas on cardiovascular response in healthy young volunteers | Wrong patient population |
| 77 | Saboo 2022 | Effect of a 6-month yoga intervention on heart rate variability among pre-diabetics | Wrong patient population |
| 78 | Santaella 2011 | Yoga respiratory training improves respiratory function and cardiac sympathovagal balance in elderly subjects: a randomised controlled trial | Wrong patient population |
| 79 | Shapira 2022 | Biomarker Response to Mindfulness Intervention in Veterans Diagnosed with Post-traumatic Stress Disorder | Wrong patient population |
| 80 | Sharma 2013 | Effect of fast and slow pranayama on perceived stress and cardiovascular parameters in young health-care students | Wrong patient population |
| 81 | Sharma 2015 | A randomized controlled pilot study of the therapeutic effects of yoga in people with Parkinson's disease | Wrong patient population |
| 82 | Sharma 2021 | Assessment of the Effect of Therapy & on Prehypertension - A clinical study | Wrong patient population |
| 83 | Shete 2017 | Effect of yoga training on inflammatory cytokines and C-reactive protein in employees of small-scale industries | Wrong patient population |
| 84 | Singh 2011 | Effects of 6-week yogic exercises training on blood pressure | Wrong patient population |
| 85 | Singh 2012 | Effect of Yoga Nidra on physiological variables in patients of menstrual disturbances of reproductive age group | Wrong patient population |
| 86 | Singh 2022 | A study protocol for a randomised controlled trial on the efficacy of yoga as an adjuvant therapy for patients with Ankylosing spondylitis amidst COVID-19 pandemic | Wrong patient population |
| 87 | Sinha 2007 | Improvement of glutathione and total antioxidant status with yoga | Wrong patient population |
| 88 | Sivaraman 2023 | EFFECT OF YOGIC PRACTICES ON PHYSIOLOGICAL VARIABLES AMONG ADULT MEN SUFFERING WITH SINUSITIS | Wrong patient population |
| 89 | Steffen 2015 | A Brief Mindfulness Exercise Reduces Cardiovascular Reactivity During a Laboratory Stressor Paradigm | Wrong patient population |
| 90 | Struh√ÅR 2019 | Effects of whole-body electrostimulation and acroyoga based exercise programme on blood pressure in a group of young women | Wrong patient population |
| 91 | Telles 2014 | Blood pressure and heart rate variability during yoga-based alternate nostril breathing practice and breath awareness | Wrong patient population |
| 92 | Thokchom 2018 | Effects of yogic intervention on pulmonary functions and health status in patients of COPD and the possible mechanisms | Wrong patient population |
| 93 | Tomar 2011 | EFFECT OF UJJAYI PRANAYAMA ON SELECTED PHYSIOLOGICAL VARIABLES | Wrong patient population |
| 94 | Voege 2014 | The Effects of a Yogic Breath Meditation Intervention on Attention Control and other Domains of Self-Control | Wrong patient population |
| 95 | Vogler 2011 | The impact of a short-term iyengar yoga program on the health and well-being of physically inactive older adults | Wrong patient population |
| 96 | Wahlstrom 2017 | Effects of yoga in patients with paroxysmal atrial fibrillation - a randomized controlled study | Wrong patient population |
| 97 | Wahlstrom 2020 | MediYoga as a part of a self-management programme among patients with paroxysmal atrial fibrillation - a randomised study | Wrong patient population |
| 98 | Walsh 2014 | Effects of a short-term mindfulness intervention on depression and immune function | Wrong patient population |
| 99 | Wilson 2022 | Fostering emotional self-regulation in female teachers at the public teaching network: A mindfulness-based intervention improving psychological measures and inflammatory biomarkers | Wrong patient population |
| 100 | Zeidan 2010 | Effects of brief and sham mindfulness meditation on mood and cardiovascular variables | Wrong patient population |
| 101 | Adhikari 2022 | A feasibility study on yoga's mechanism of action for chronic low back pain: psychological and neurophysiological changes, including global gene expression and DNA methylation, following a yoga intervention for chronic low back pain | Wrong study design |
| 102 | Agte 2011 | The effects of Sudarshan Kriya Yoga on some physiological and biochemical parameters in mild hypertensive patients | Wrong study design |
| 103 | Altman 2001 | A brief therapy model to reduce stress by practicing breathing exercises, mindful meditation, and yoga stretching | Wrong study design |
| 104 | Arul 2020 | Influence Of Varied Yogasanas On Selected Biochemical Variables | Wrong study design |
| 105 | Bagga 2015 | Lipid peroxidation and antioxidant status in fibrocystic breast disease with and without Sudarshan Kriya yoga | Wrong study design |
| 106 | Bal 2015 | Effects of Surya Nadi Pranayama (Right Nostril Breathing) on Hematological Parameters | Wrong study design |
| 107 | Bal 2015 | Effects of Chandra-Nadi Pranayama on metabolic fitness and bone integrity | Wrong study design |
| 108 | Balaji 2011 | THE IMPACT OF HATHA YOGA PRACTICES AND SURYA NAMASKAR FOR DEVELOPING WOMEN PHYSIOLOGICAL PARAMETERS | Wrong study design |
| 109 | Barrientos 2021 | Effects of Hatha-Vinyasa Yoga practice on the autonomic regulation and perceived pain of older women living in high southern latitude. A pilot study | Wrong study design |
| 110 | Bhatt 2019 | The role of yoga therapy in the management of bronchial asthma (tamaka shwasa) | Wrong study design |
| 111 | Bisht 2018 | Sperm DNA damage: consequences of the impact of yogic cognitive behavior practices | Wrong study design |
| 112 | Borthakur 2020 | Yoga based lifestyle intervention and its impact on depression, quality of life and cellular aging in infertile couples | Wrong study design |
| 113 | Bute 2011 | A comparative study of antioxidant status in yoga and normal adult males | Wrong study design |
| 114 | Chattopadhyay 2020 | Yoga programme for type-2 diabetes prevention (YOGA-DP) among high risk people in India: A multicentre feasibility randomised controlled trial protocol | Wrong study design |
| 115 | Chaturvedi 2016 | Comparative assessment of the effects of hatha yoga and physical exercise on biochemical functions in perimenopausal women | Wrong study design |
| 116 | Chaturvedi 2020 | Effect of yoga therapy on state and trait anxiety in perimenopausal women: A non-randomized controlled study | Wrong study design |
| 117 | Hunter 2013 | The effect of bikram yoga on arterial stiffness in young and older adults | Wrong study design |
| 118 | Kim 2005 | Yoga for Prevention of Cardiovascular Diseases | Wrong study design |
| 119 | Kim 2017 | Elderly-customized hatha yoga effects on the vascular inflammation factors of elderly women | Wrong study design |
| 120 | Lau 2015 | Effects of a 12-Week Hatha Yoga Intervention on Metabolic Risk and Quality of Life in Hong Kong Chinese Adults with and without Metabolic Syndrome | Wrong study design |
| 121 | Luo 2020 | Effect of yoga combined with aerobic exercise intervention on morphological and blood lipid indicators in female college students | Wrong study design |
| 122 | Manchanda 2014 | Yoga - A promising technique to control cardiovascular disease | Wrong study design |
| 123 | Mayor 2014 | Yoga reduces cardiovascular risk as much as walking or cycling, study shows | Wrong study design |
| 124 | Pal 2014 | Age-related changes in cardiovascular system, autonomic functions, and levels of BDNF of healthy active males: role of yogic practice | Wrong study design |
| 125 | Porwal 2022 | Assessment of impact of Yoga exercises on releasing neurological stress | Wrong study design |
| 126 | Ramkumar 2014 | Poster Session 3 ‚Äì Afternoon | Wrong study design |
| 127 | Saboo 2021 | Effect of Six Months Yoga Intervention on Metabolic Profile and Carotid Intima Media Thickness in Prediabetes | Wrong study design |
| 128 | Saboo 2023 | A Study to Assess and Correlate Metabolic Parameters with Carotid Intima-Media Thickness after Combined Approach of Yoga Therapy among Prediabetics | Wrong study design |
| 129 | Sen 2020 | Yoga could reduce the burden and symptoms of atrial fibrillation as well as medication related side effects and the complications with cardiac ablation | Wrong study design |
| 130 | Smith 2013 | Abstracts of the 14th International Congress on Schizophrenia Research (ICOSR). April 21-15, 2013. Orlando Grande Lakes, Florida, USA | Wrong study design |
| 131 | Suganthi 2023 | Effect of Simplifed Kundalini Yoga Practices on Haematological and Hormonal Variables among Medical Students | Wrong study design |
| 132 | Sujatha 2020 | Impact of yogic practices on physiological selected factors of overweight adult women | Wrong study design |

Manual Searches

| 1 | Chauhan A., Semwal D.K., Mishra S.P., Semwal R.B.: Yoga practice improves the body mass index and blood pressure: a randomized controlled trial. Int J Yoga 2017; 10: pp. 103-106. | Already been included from database searches |
| --- | --- | --- |
| 2 | Cramer H., Thoms M.S., Anheyer D., Lauche R., Dobos G.: Yoga in women with abdominal obesity: a randomized controlled trial. Dtsch Arztebl Int 2016; 113: pp. 645-652. | Already been included from database searches |
| 3 | Hegde S.V., Adhikari P., Shetty S., Manjrekar P., D'Souza V.: Effect of community-based yoga intervention on oxidative stress and glycemic parameters in prediabetes: a randomized controlled trial. Complement Ther Med 2013; 21: pp. 571-576. | Already been included from database searches |
| 4 | Hewett ZL, Pumpa KL, Smith CA, Fahey PP, Cheema BS Effect of a 16-week Bikram yoga program on heart rate variability and associated cardiovascular disease risk factors in stressed and sedentary adults: a randomized controlled trial | Already been included from database searches |
| 5 | Hunter SD, Laosiripisan J, Elmenshawy A, Tanaka H Effects of yoga interventions practised in heated and thermoneutral conditions on endothelium-dependent vasodilatation: the Bikram yoga heart study | Already been included from database searches |
| 6 | Jabir P.K., Sadananda B., Das K.S.: Effect of Balasana on cardiac parameters among healthy medical students. Natl J Physiol Pharm Pharmacol 2017; 7: pp. 1342-1346. | Already been included from database searches |
| 7 | Kaur N, Majumdar V, Nagarathna R, Malik N, Anand A, Nagendra HR Diabetic yoga protocol improves glycemic, anthropometric and lipid levels in high risk individuals for diabetes: a randomized controlled trial from Northern India | Already been included from database searches |
| 8 | Kim S., Bemben M.G., Bemben D.A.: Effects of an 8-month yoga intervention on arterial compliance and muscle strength in premenopausal women. J Sports Sci Med 2012; 11: pp. 322-330. | Already been included from database searches |
| 9 | Lee JA, Kim JW, Kim DY Effects of yoga exercise on serum adiponectin and metabolic syndrome factors in obese postmenopausal women | Already been included from database searches |
| 10 | McDermott KA, Rao MR, Nagarathna R, Murphy EJ, Burke A, Nagendra RH, et al A yoga intervention for type diabetes risk reduction: a pilot randomized controlled trial | Already been included from database searches |
| 11 | Thiyagarajan R, Pal P, Pal GK, Subramanian SK, Trakroo M, Bobby Z, et al Additional benefit of yoga to standard lifestyle modification on blood pressure in prehypertensive subjects: a randomized controlled study | Already been included from database searches |
| 12 | Yang et al.Utilization of 3-Month Yoga Program for Adults at High Risk for Type Diabetes: A Pilot Study | Already been included from database searches |
| 13 | Ruby M, Repka CP, Arciero PJ Comparison of protein-pacing alone or with yoga/stretching and resistance training on glycemia, total and regional body composition, and aerobic fitness in overweight women | Wrong comparator |
| 14 | Telles S, Sharma SK, Yadav A, Singh N, Balkrishna A A comparative controlled trial comparing the effects of yoga and walking for overweight and obese adults | Wrong comparator |
| 15 | Bowman AJ, Clayton RH, Murray A et al | Wrong comparison |
| 16 | Fields JZ, Walton KG, Schneider RH et al | Wrong intervention |
| 17 | 5Tillin T, Tuson C, Sowa B, Chattopadhyay K, Sattar N, Welsh P, et al Yoga and cardiovascular health trial (YACHT): a UK-based randomised mechanistic study of a yoga intervention plus usual care versus usual care alone following an acute coronary event | Wrong patient population |
| 18 | Agte VV Sudarshan kriya yoga for treating type diabetes | Wrong patient population |
| 19 | Arumugam G, Nagarathna R, Majumdar V, Singh M, Srinivasalu R, Sanjival R, et al Yoga-based lifestyle treatment and composite treatment goals in Type diabetes in a rural South Indian setup- a retrospective study | Wrong patient population |
| 20 | Bagga O.P., Gandhi A.: A comparative study of the effect of Transcendental Meditation (T.M.) and Shavasana practice on cardiovascular system. Indian Heart J 1983; 35: pp. 39-45. | Wrong patient population |
| 21 | Bal B.S.: Effects of Chandra-Nadi Pranayama on metabolic fitness and bone integrity. Medicina Sportiva 2015; 11: pp. 2621-2627. | Wrong patient population |
| 22 | Balaji PA, Varne SR, Sadat-Ali S Effects of yoga pranayama practices on metabolic parameters and anthropometry in type diabetes | Wrong patient population |
| 23 | Bidwell A.J., Yazel B., Davin D., Fairchild T.J., Kanaley J.A.: Yoga training improves quality of life in women with asthma. J Altern Complement Med 2012; 18: pp. 749-755. | Wrong patient population |
| 24 | Bindra M, Seema D, Shema N Influence of pranayamas and yoga-asanas on blood glucose, lipid profile and hba1c in type diabetes 2013 | Wrong patient population |
| 25 | Biswas D Effect of relaxation technique on lipid profile in cases of essential hypertension | Wrong patient population |
| 26 | Blumenthal JA, Emery CF, Madden DJ, Coleman RE, Riddle MW, Schniebolk S, et al Effects of exercise training on cardiorespiratory function in men and women older than years of age | Wrong patient population |
| 27 | Bower J., Greendale G., Crosswell A., Garet D., Sternlieb B., Ganz P.…Cole S. (2014). Yoga reduces inflammatory signaling in fatigued breast cancer survivors: A randomized controlled trial. | Wrong patient population |
| 28 | Cade WT, Reeds DN, Mondy KE, Overton ET, Grassino J, Tucker S, et al Yoga lifestyle intervention reduces blood pressure in HIV-infected adults with cardiovascular disease risk factors | Wrong patient population |
| 29 | Chen et al.Effects of 8-Week Hatha Yoga Training on Metabolic and Inflammatory Markers in Healthy, Female Chinese Subjects: A Randomized Clinical Trial | Wrong patient population |
| 30 | Cohen BE, Chang AA, Grady D, Kanaya AM Restorative yoga in adults with metabolic syndrome: a randomized, controlled pilot trial | Wrong patient population |
| 31 | Cohen DL Bloedon LT Rothman RL Farrar JT Galantino ML Volger S Mayor C Szapary PO Townsend RR . Iyengar yoga versus enhanced usual care on blood pressure in patients with prehypertension to stage I hypertension: a randomized controlled trial. Evid Based Complement Alternat Med2011; 2011:546428. | Wrong patient population |
| 32 | Cusumano JA, Robinson SE | Wrong patient population |
| 33 | Deepa T., Sethu G., Thirrunavukkarasu N.: Effect of yoga and meditation on mild to moderate essential hypertensives. J Clin Diagn Res 2012; 6: pp. 21-26. | Wrong patient population |
| 34 | Dinesh T., Venkatesan R., Venkidusamy S.: Effect of 12 weeks of pranayama training on basal physiological parameters in young, healthy volunteers. Panacea Journal of Medical Science 2014; 4: pp. 28-30. | Wrong patient population |
| 35 | Dutta A, Green SR, Balayogi AB, Hemachandar R, Dhivya P, Mathew KT Effect of yoga therapy on fasting lipid profile in chronic kidney disease: a comparative study | Wrong patient population |
| 36 | Gopal A., Mondal S., Gandhi A., Arora S., Bhattacharjee J.: Effect of integrated yoga practices on immune responses in examination stress - a preliminary study. Int J Yoga 2011; 4: pp. 26-32. | Wrong patient population |
| 37 | Gordon L, McGrowder DA, Pena YT, Cabrera E, Lawrence-Wright M Effect of exercise therapy on lipid parameters in patients with end-stage renal disease on hemodialysis | Wrong patient population |
| 38 | Gordon LA, Morrison EY, McGrowder DA, Young R, Fraser YT, Zamora EM, et al Effect of exercise therapy on lipid profile and oxidative stress indicators in patients with type diabetes | Wrong patient population |
| 39 | Gupta U, Gupta Y, Jose D, Mani K, Jyotsna VP, Sharma G, et al Effectiveness of yoga-based exercise program compared to usual care, in improving HbA1c in individuals with type diabetes: a randomized control trial | Wrong patient population |
| 40 | Haber D.: Yoga as a preventive health care program for white and black elders: an exploratory study. Int J Aging Hum Dev 1983; 17: pp. 169-176. | Wrong patient population |
| 41 | Habibi N., Farsani Z.H., Yazdani B., Arianshakib R., Noruozi P.: The influence of yoga-on risk profiles programs in women with diabetes type II. Adv Environ Biol 2013; 2013: pp. 550-556. | Wrong patient population |
| 42 | Hagins M Rundle A Consedine NS Khalsa SB . A randomized controlled trial comparing the effects of yoga with an active control on ambulatory blood pressure in individuals with prehypertension and stage 1 hypertension. J Clin Hypertens (Greenwich)2014; 16:54–62. | Wrong patient population |
| 43 | Harinath K, Malhotra AS, Pal K et al | Wrong patient population |
| 44 | Harkess K. N., Ryan J., Delfabbro P. H., Cohen-Woods S. (2016). Preliminary indications of the effect of a brief yoga intervention on markers of inflammation and DNA methylation in chronically stressed women. | Wrong patient population |
| 45 | Harris A.R., Jennings P.A., Katz D.A., Abenavoli R.M., Greenberg M.T.: Promoting stress management and wellbeing in educators: feasibility and efficacy of a school-based yoga and mindfulness intervention. Mindfulness 2016; 7: pp. 143-154. | Wrong patient population |
| 46 | Hegde S.V., Adhikari P., Subbalakshmi N.K., Nandini M., Rao G.M., D'Souza V.: Diaphragmatic breathing exercise as a therapeutic intervention for control of oxidative stress in type 2 diabetes mellitus. Complement Ther Clin Pract 2012; 18: pp. 151-153. | Wrong patient population |
| 47 | Innes K.E., Selfe T.K.: The effects of a gentle yoga program on sleep, mood, and blood pressure in older women with Restless Legs Syndrome (RLS): a preliminary randomized controlled trial. Evid Based Complement Alternat Med 2012; 2012: pp. 294058. | Wrong patient population |
| 48 | Kanaya AM, Araneta MR, Pawlowsky SB, Barrett-Connor E, Grady D, Vittinghoff E, et al Restorative yoga and metabolic risk factors: the practicing restorative yoga vs | Wrong patient population |
| 49 | Kanojia S., Sharma V.K., Gandhi A., Kapoor R., Kukreja A., Subramanian S.K.: Effect of yoga on autonomic functions and psychological status during both phases of menstrual cycle in young healthy females. J Clin Diagn Res 2013; 7: pp. 2133-2139. | Wrong patient population |
| 50 | Kiecolt-Glaser J. K., Bennett J. M., Andridge R., Peng J., Shapiro C. L., Malarkey W. B.…Glaser R. (2014). Yoga’s impact on inflammation, mood, and fatigue in breast cancer survivors: A randomized controlled trial. | Wrong patient population |
| 51 | Kim HN, Ryu J, Kim KS, Song SW Effects of yoga on sexual function in women with metabolic syndrome: a randomized controlled trial | Wrong patient population |
| 52 | Krishna B.H., Pal P., Pal G.K., et. al.: Effect of yoga therapy on heart rate, blood pressure and cardiac autonomic function in heart failure. J Clin Diagn Res 2014; 8: pp. 14-16. | Wrong patient population |
| 53 | Kumpatla S, Michael C, Viswanathan V Effect of yogasanas on glycaemic, haemodynamic and lipid profile in newly diagnosed subjects with type diabetes | Wrong patient population |
| 54 | Lakkireddy D., Atkins D., Pillarisetti J., et. al.: Effect of yoga on arrhythmia burden, anxiety, depression, and quality of life in paroxysmal atrial fibrillation: the YOGA My Heart Study. J Am Coll Cardiol 2013; 61: pp. 1177-1182. | Wrong patient population |
| 55 | Latha Kaliappan KV . Yoga, Pranayama, thermal biofeedback techniques in the management of stress and high blood pressure. J Ind Psychol1991; 9: 36–46. | Wrong patient population |
| 56 | Lathadevi G.V., Maheswari T.U., Nagashree R.: Modulation of cardiovascular response after ujjayi pranayama and shavasana training in normal human volunteers. J Clin Diagn Res 2012; 6: pp. 571-573. | Wrong patient population |
| 57 | lau et al.Effects of a 12-Week Hatha Yoga Intervention on Metabolic Risk and Quality of Life in Hong Kong Chinese Adults with and without Metabolic Syndrome | Wrong patient population |
| 58 | Madanmohan , Mahadevan S.K., Balakrishnan S., Gopalakrishnan M., Prakash E.S.: Effect of six weeks yoga training on weight loss following step test, respiratory pressures, handgrip strength and handgrip endurance in young healthy subjects. Indian J Physiol Pharmacol 2008; 52: pp. 164-170. | Wrong patient population |
| 59 | Malathi A., Damodaran A., Shah N., Krishnamurthy G., Namjoshi P., Ghodke S.: Psychophysiological changes at the time of examination in medical students before and after the practice of yoga and relaxation. Indian J Psychiatry 1998; 40: pp. 35-40. | Wrong patient population |
| 60 | Manna I Effects of yoga training on body composition, cardiovascular and biochemical parameters in healthy adult male volunteers | Wrong patient population |
| 61 | McCaffrey R., Ruknui P., Hatthakit U., Kasetsomboon P.: The effects of yoga on hypertensive persons in Thailand. Holist Nurs Pract 2005; 19: pp. 173-180. | Wrong patient population |
| 62 | Misra P, Sharma G, Tandon N, Kant S, Sangral M, Rai SK, et al Effect of community-based structured yoga program on Hba1c level among type diabetes mellitus patients: an interventional study | Wrong patient population |
| 63 | Mizuno J, Monteiro HL An assessment of a sequence of yoga exercises to patients with arterial hypertension | Wrong patient population |
| 64 | Mondal S, Kundu B, Saha S Yoga as a therapeutic intervention for the management of type diabetes mellitus | Wrong patient population |
| 65 | Mourya M Mahajan AS Singh NP Jain AK . Effect of slow- and fast-breathing exercises on autonomic functions in patients with essential hypertension. J Altern Complement Med2009; 15:711–717. | Wrong patient population |
| 66 | Murthy SN, Rao NS, Nandkumar B, Kadam A Role of naturopathy and yoga treatment in the management of hypertension | Wrong patient population |
| 67 | Murugesan R., Govindarajulu N., Bera T.: Effect of selected yogic practices on the management of hypertension. Indian J Physiol Pharmacol 2000; 44: pp. 207-210. | Wrong patient population |
| 68 | Nagarathna et al.Effectiveness of Yoga Lifestyle on Lipid Metabolism in a Vulnerable Population—A Community Based Multicenter Randomized Controlled Trial | Wrong patient population |
| 69 | Nagarathna R Efficacy of yoga based life style modification program on medication score and lipid profile in type diabetes-a randomized control study | Wrong patient population |
| 70 | Nelson D., Reed J., Buck S.M.: Effect of a 16-week yoga program on blood pressure in healthy college students. Physical Educator 2014; 71: pp. 533-544. | Wrong patient population |
| 71 | Pal A, Srivastava N, Tiwari S, Verma NS, Narain VS, Agrawal GG, et al Effect of yogic practices on lipid profile and body fat composition in patients of coronary artery disease | Wrong patient population |
| 72 | Papp M.E., Lindfors P., Nygren-Bonnier M., Gullstrand L., Wändell P.E.: Effects of high-intensity Hatha yoga on cardiovascular fitness, adipocytokines, and apolipoproteins in healthy students: a randomized controlled study. [published correction appears in J Altern Complement Med . 2017;23(5):396] J Altern Complement Med 2016; 22: pp. 81-87. | Wrong patient population |
| 73 | Prasad A, Bhardwaj S, Dwivedi S, Arora YK, Sharma V Effect of yoga in post-myocardial infarction cases | Wrong patient population |
| 74 | Priya J.V., Kanniammal C., Mahendra J., Valli G.: Impact of yoga on blood pressure and quality of life in patients with hypertension. Int J Pharmaceut Clin Res 2017; 9: pp. 413-416. | Wrong patient population |
| 75 | Pullen P. R., Nagamia S. H., Mehta P. K., Thompson W. R., Benardot D., Hammoud R.…Khan B. V. (2008). Effects of yoga on inflammation and exercise capacity in patients with chronic heart failure. | Wrong patient population |
| 76 | Pullen P. R., Thompson W. R., Benardot D., Brandon L. J., Mehta P. K., Rifai L.…Khan B. V. (2010). Benefits of yoga for African American heart failure patients | Wrong patient population |
| 77 | Rachiwong S., Panasiriwong P., Saosomphop J., Widjaja W., Ajjimaporn A.: Effects of modified Hatha yoga in industrial rehabilitation on physical fitness and stress of injured workers. J Occup Rehabil 2015; 25: pp. 669-674. | Wrong patient population |
| 78 | Raghuram N, Parachuri VR, Swarnagowri MV, Babu S, Chaku R, Kulkarni R, et al Yoga based cardiac rehabilitation after coronary artery bypass surgery: one-year results on LVEF, lipid profile and psychological states–a randomized controlled study | Wrong patient population |
| 79 | Rajbhoj P. H., Shete S. U., Verma A., Bhogal R. S. (2015). Effect of yoga module on pro-inflammatory and anti-inflammatory cytokines in industrial workers of Lonavla: A randomized controlled trial. | Wrong patient population |
| 80 | Rani KB, Sreekumaran E Yogic practice and diabetes mellitus in geriatric patients | Wrong patient population |
| 81 | Rao R. M., Vadiraja H., Nagaratna R., Gopinath K., Patil S., Diwakar R. B.…Nagendra H. (2017). Effect of yoga on sleep quality and neuroendocrine immune response in metastatic breast cancer patients. | Wrong patient population |
| 82 | Ray U.S., Mukhopadhyaya S., Purkayastha S.S., et. al.: Effect of yogic exercises on physical and mental health of young fellowship course trainees. Indian J Physiol Pharmacol 2001; 45: pp. 37-53. | Wrong patient population |
| 83 | Ray US, Sinha B, Tomer OS et al | Wrong patient population |
| 84 | Roche L.T., Hesse B.M.: Application of an integrative yoga therapy programme in cases of essential arterial hypertension in public healthcare. Complement Ther Clin Pract 2014; 20: pp. 285-290. | Wrong patient population |
| 85 | Saptharishi L., Soudarssanane M., Thiruselvakumar D., et. al.: Community-based randomized controlled trial of non-pharmacological interventions in prevention and control of hypertension among young adults. Indian J Community Med 2009; 34: pp. 329-334. | Wrong patient population |
| 86 | Shantakumari et al.Effects of a yoga intervention on lipid profiles of diabetes patients with dyslipidemia | Wrong patient population |
| 87 | Sharma KNS, Pailoor S, Choudhary NR, Bhat P, Shrestha S Integrated yoga practice in cardiac rehabilitation program: a randomized control trial | Wrong patient population |
| 88 | Sharma S Influence of yoga on status of lipid indices in type diabetes mellitus subjects | Wrong patient population |
| 89 | Shete SU, Verma A, Kulkarni DD, Bhogal RS Effect of yoga training on inflammatory cytokines and C-reactive protein in employees of small-scale industries | Wrong patient population |
| 90 | Shetty KG Combined effect of yoga and naturopathy treatments on lipid profile among type diabetes mellitus -a randomised trail | Wrong patient population |
| 91 | Singh AK, Kaur N, Kaushal S, Tyagi R, Mathur D, Sivapuram MS, et al Partitioning of radiological, stress and biochemical changes in pre-diabetic women subjected to diabetic yoga protocol | Wrong patient population |
| 92 | Singh S, Kyizom T, Singh KP, Tandon OP, Madhu SV Influence of pranayamas and yoga-asanas on serum insulin, blood glucose and lipid profile in type diabetes | Wrong patient population |
| 93 | Singh S., Gaurav V., Parkash V.: Effects of a 6-week nadi-shodhana pranayama training on cardio-pulmonary parameters. J Phys Educ Sport Manag 2011; 2: pp. 44-47. | Wrong patient population |
| 94 | Singh V.K., Bhandari R.B., Rana B.B.: Effect of yogic package on rheumatoid arthritis. Indian J Physiol Pharmacol 2011; 55: pp. 329-335. | Wrong patient population |
| 95 | Siu P.M., Yu A.P., Benzie I.F., Woo J.: Effects of 1-year yoga on cardiovascular risk factors in middle-aged and older adults with metabolic syndrome: a randomized trial. Diabetol Metab Syndr 2015; 7: pp. 40. | Wrong patient population |
| 96 | Siu PM, Yu AP, Benzie IF, Woo J Effects of 1-year yoga on cardiovascular risk factors in middle-aged and older adults with metabolic syndrome: a randomized trial | Wrong patient population |
| 97 | Sivapuram MS, Srivastava V, Kaur N, Anand A, Nagarathna R, Patil S, et al Ayurveda body-mind constitutional types and role of yoga intervention among type diabetes mellitus population of Chandigarh and Panchkula regions | Wrong patient population |
| 98 | Sohl S. J., Danhauer S. C., Birdee G. S., Nicklas B. J., Yacoub G., Aklilu M., Avis N. E. (2016). A brief yoga intervention implemented during chemotherapy: A randomized controlled pilot study. | Wrong patient population |
| 99 | Stachenfeld NS, Mack GW, DiPietro L et al | Wrong patient population |
| 100 | Subramanian S, Elango T, Malligarjunan H, Kochupillai V, Dayalan H Role of sudarshan kriya and pranayam on lipid profile and blood cell parameters during exam stress: a randomized controlled trial | Wrong patient population |
| 101 | Sujatha T., Judie A.: Effectiveness of a 12-week yoga program on physiopsychological parameters in patients with hypertension. Int J Pharmaceut Clin Res 2014; 6: pp. 329-335. | Wrong patient population |
| 102 | Tracy BL, Hart CE | Wrong patient population |
| 103 | Vaishali K Effects of yoga-based program on glycosylated hemoglobin level serum lipid profile in community dwelling elderly subjects with chronic type diabetes mellitus–a randomized controlled trial | Wrong patient population |
| 104 | Viswanathan V, Sivakumar S, Sai Prathiba A, Devarajan A, George L, Kumpatla S Effect of yoga intervention on biochemical, oxidative stress markers, inflammatory markers and sleep quality among subjects with type diabetes in South India: results from the SATYAM project | Wrong patient population |
| 105 | Vogler J., O'Hara L., Gregg J., Burnell F.: The impact of a short-term iyengar yoga program on the health and well-being of physically inactive older adults. Int J Yoga Therap 2011; 21: pp. 61-72. | Wrong patient population |
| 106 | Wolever R.Q., Bobinet K.J., McCabe K., et. al.: Effective and viable mind-body stress reduction in the workplace: a randomized controlled trial. J Occup Health Psychol 2012; 17: pp. 246-258. | Wrong patient population |
| 107 | Wolff M, Memon AA, Chalmers JP, Sundquist K, Midlöv P Yoga’s effect on inflammatory biomarkers and metabolic risk factors in a high risk population - a controlled trial in primary care | Wrong patient population |
| 108 | Wolff M., Rogers K., Erdal B., Chalmers J.P., Sundquist K., Midlöv P.: Impact of a short home-based yoga programme on blood pressure in patients with hypertension: a randomized controlled trial in primary care. J Hum Hypertens 2016; 30: pp. 599-605. | Wrong patient population |
| 109 | Wolff M., Sundquist K., Larsson Lönn S., Midlöv P.: Impact of yoga on blood pressure and quality of life in patients with hypertension—a controlled trial in primary care, matched for systolic blood pressure. BMC Cardiovasc Disord 2013; 13: pp. 111. | Wrong patient population |
| 110 | Yadav R, Yadav RK, Khadgawat R, Pandey RM, Upadhyay AD, Mehta N Randomized controlled trial of a 12-week yoga-based (including diet) lifestyle vs | Wrong patient population |
| 111 | Yurtkuran M, Alp A, Yurtkuran M, Dilek K A modified yoga-based exercise program in hemodialysis patients: a randomized controlled study | Wrong patient population |
| 112 | Chen K.M., Chen M.H., Hong S.M., Chao H.C., Lin H.S., Li C.H.: Physical fitness of older adults in senior activity centres after 24-week silver yoga exercises. J Clin Nurs 2008; 17: pp. 2634-2646. | Wrong study design |
| 113 | Cho H. K., Moon W., Kim J. (2015). Effects of yoga on stress and inflammatory factors in patients with chronic low back pain: A non-randomized controlled study. | Wrong study design |
| 114 | Parma D. L., Hughes D. C., Ghosh S., Li R., Treviño-Whitaker R. A., Ogden S. M., Ramirez A. G. (2015). Effects of six months of yoga on inflammatory serum markers prognostic of recurrence risk in breast cancer survivors. | Wrong study design |
| 115 | Sarvottam K., Magan D., Yadav R. K., Mehta N., Mahapatra S. C. (2013). Adiponectin, interleukin-6, and cardiovascular disease risk factors are modified by a short-term yoga-based lifestyle intervention in overweight and obese men. | Wrong study design |
| 116 | Yadav R. K., Magan D., Mehta N., Sharma R., Mahapatra S. C. (2012). Efficacy of a short-term yoga-based lifestyle intervention in reducing stress and inflammation: Preliminary results. | Wrong study design |
| 117 | Chauhan, Shweta; Patra, Sanjib; Singh, Shailendra Pratap; Lakhani, Jitendra D..2023.Combined effect of yoga and naturopathy in uncomplicated varicose vein disease - a prospective randomized controlled trial | Wrong comparison |
| 118 | Sen, N.; Tanwar, S.; Kulshreshtra, A..2023.Better Reduction of Blood Pressure with Combined Integrated Indian Yoga And Chinese Aerobics Versus Indian Yoga or Chinese Aerobic Exercise Alone | Wrong comparison |
| 119 | Colvin, A.; Murray, L.; Noble, J.; Chastin, S..2024.Effects of Breaking Up Sedentary Behavior With Short Bouts of Yoga and Tai-Chi on Glycemia, Concentration, and Well-Being | Wrong intervention |
| 120 | Guha, N.; Ghosh, S.; Mandal, S.; Das, A. D.; Palanisamy, C.; Maiti, S.; Ghosh, P.; Singh, N. K.; Koley, M.; Saha, S..2023.A Double-Blind, Randomized, Placebo-Controlled Clinical Trial to Evaluate the Efficacy of Individualized Homeopathic Medicines in Pre-diabetes | Wrong intervention |
| 121 | Zhu, D.; Jiang, M.; Xu, D.; Sch√∂llhorn, W. I..2020.Long-Term Effects of Mind-Body Exercises on the Physical Fitness and Quality of Life of Individuals With Substance Use Disorder-A Randomized Trial | Wrong intervention |
| 122 | Unick, J. L.; Dunsiger, S. I.; Bock, B. C.; Sherman, S. A.; Braun, T. D.; Hayes, J. F.; Goldstein, S. P.; Wing, R. R..2023.A randomized trial examining the effect of yoga on dietary lapses and lapse triggers following behavioral weight loss treatment | Wrong outcomes |
| 123 | Badri, K.; Subbulakshmi, V.; Elayaraja, M.; Gogoi, H.; Govindasamy, K..2024.Impact of Yoga Therapy on D-dimer Level in Asymptomatic Varicose Vein Disease ‚Äì a Randomized Controlled Trial | Wrong population |
| 124 | Ebnezar, J.; Nagarathna, R.; Yogitha, B.; Nagendra, H. R..2012.Effect of integrated yoga therapy on pain, morning stiffness and anxiety in osteoarthritis of the knee joint: a randomized control study | Wrong population |
| 125 | Gupt, A. M.; Madaik, T. S.; Agarwal, A.; Rajta, P. N..2024.Impact of Meditation and Pranayama on Autonomic Nervous System Balance | Wrong population |
| 126 | Haag, F. B.; Resende, e Silva D. T.; Antunes, C. S.; Waclawovsky, G.; Lucchese-Lobato, F..2024.Effects of circuit training and Yoga on biochemical and psychological responses to stress and cardiovascular markers: a randomized clinical trial with nursing and medical students in Southern Brazil | Wrong population |
| 127 | Krishna, B. H.; Pulaganti, M.; Sekhar, A. C.; Jampala, S..2023.EXPLORING THE EFFECTS OF YOGA THERAPY ON CARDIOVASCULAR RISK PROFILE, CARDIAC WORKLOAD, AND OXYGEN DEMAND IN INDIVIDUALS WITH PREHYPERTENSION: a PILOT STUDY | Wrong population |
| 128 | Kumar, M.; Singh, R. K.; Kumari, S..2024.Effect of Yoga on Pulse Rate and Blood Pressure in Medical Student of Darbhanga Medical College, Laheriasarai, Bihar | Wrong population |
| 129 | Malik, S. K.; Tayal, N.; Singh, H.; Gourh, S.; Kumar, A..2023.To Study the Role of Yoga in Physical Wellbeing and COVID Antibody Production in COVID Vaccine Recipients | Wrong population |
| 130 | Mathew, Deepak; Rangasamy, Muthulakshmi.2024.Effect of Yoga Therapy on Insomnia Severity and Systolic Blood Pressure in Aged Women: A 12-Week Intervention Study Conducted in Kerala | Wrong population |
| 131 | Moreno, S.; Becerra, L.; Ortega, G.; Suarez-Orteg√≥n, M. F.; Moreno, F..2023.Effect of Hatha Yoga and meditation on academic stress in medical students‚ÄîClinical trial | Wrong population |
| 132 | Narnolia, P. K.; Binawara, B. K.; Mehra, M.; Sharma, P.; Sharma, S..2024.To Study the Effect of Sudarshan Kriya Yoga and Conventional Physical Exercise on Blood Pressure, Pulse Rate and Quality of Life in Prehypertensive Subjects | Wrong population |
| 133 | Narnolia, P. K.; Binawara, B. K.; Mehra, M.; Vyas, P..2024.Effect of Sudarshan Kriya Yogaand Physical Exercise on Fasting Blood Sugar and Perceived Stress in Prediabetic Subjects | Wrong population |
| 134 | √ñZaras √ñZ, G..2023.Effect of Laughter Yoga on Anxiety, Depression and Physiological Parameters in Nursing Students During the COVID-19 Pandemic: a Randomized Controlled Study | Wrong population |
| 135 | Patil, Y.; Sabbu, K.; Iyer, R. B.; Philip, S. T.; Armila Nadhar, A.; Thakur, K. S.; Kadu, P.; Thakur, M..2024.Effect of Heartfulness Meditation on Oxidative Stress and Mindfulness in Healthy Participants | Wrong population |
| 136 | Rao, Aayushee; Kacker, Sudhanshu; Saboo, Neha.2023.A Study to Evaluate the Effect of a Combined Approach of Yoga and Diet in High-risk Cardiovascular Subjects | Wrong population |
| 137 | Rao, V. S.; Murugesan, S..2024.EFFECT OF YOGA THERAPY ONFASTING BLOOD SUGAR AND HEMOGLOBIN AMONG GERIATRIC MEN | Wrong population |
| 138 | Richard, D.; Rousseau, D.; Umapathy, K.; Pandya, H.; Rousis, G.; Peeples, P..2024.Exploring the Impact of a Trauma-informed Yoga and Mindfulness Curriculum for Multiple Populations: A Pilot Study | Wrong population |
| 139 | Saboo, Neha; Kacker, Sudhanshu.2023.A Study to Assess and Correlate Metabolic Parameters with Carotid Intima-Media Thickness after Combined Approach of Yoga Therapy among Prediabetics | Wrong population |
| 140 | Saboo, N.; Kacker, S..2024.A Study on Yoga-Based Lifestyle Intervention versus Dietary Intervention Alone on Cardiometabolic Risk Factors among People with Prediabetes | Wrong population |
| 141 | Samer, S. F.; Ahmed, M. A.; Hassan, M. A..2023.Effect of yoga on endothelial function, vascular compliance and sympathetic tone in elderly subjects with increased pulse pressure | Wrong population |
| 142 | Sarshin, Amir; Arabzadeh, Ehsan; Zargani, Mehdi; Sedighi, Sahar; Tehrani, Mona Abdolhamid; Feizolahi, Foad.2023.The Effect of Three Ten-Day Courses of Yoga Exercises and Zinc Sulfate Supplementation on Prostaglandin E in Non-athletic Young Women with Primary Dysmenorrhea | Wrong population |
| 143 | Snigdha, A.; Majumdar, V.; Manjunath, N. K.; Jose, A..2024.Yoga-based lifestyle intervention for healthy ageing in older adults: a two-armed, waitlist randomized controlled trial with multiple primary outcomes | Wrong population |
| 144 | Subramanian, S.; Elango, T.; Malligarjunan, H.; Kochupillai, V.; Dayalan, H..2012.Role of sudarshan kriya and pranayam on lipid profile and blood cell parameters during exam stress: a randomized controlled trial | Wrong population |
| 145 | Talukdar, P. M.; Reddy, P. V.; Bhargav, P. H.; Subbanna, M.; Karmani, S.; Arasappa, R.; Subramanian, G. V.; Kesavan, M.; Debnath, M..2023.Long-term Add-on Yoga Therapy Modulates Oxidative Stress Pathway and Offers Clinical Benefits in Major Depressive Disorder: a Randomized Controlled Trial | Wrong population |
| 146 | Killi, A.; Damerla, U. R..2023.Effectiveness of yoga practice on the glycemic status and body mass index in pre-diabetic individuals | Wrong study design |
| 147 | Koncz, √Å; Csala, B.; K√∂rmendi, J.; Horv√°th, √Å; D√∂m√∂t√∂r, Z.; Selmeci, C.; Bogd√°n, √Å S.; K√∂teles, F.; Boros, S..2024.Effects of a complex yoga-based intervention on physical characteristics | Wrong study design |
| 148 | Pal, R.; Rao, S.; Deo, G.; Basavaraddi, I. V.; Sharma, N.; Madaan, L..2024.Impacts of the Surya Namaskar on Body Composition and Physiological Parameters among Yoga and Non Yoga Professionals: A Quasi-experimental Study | Wrong study design |
| 149 | Patel, N. S.; Patel, N. K.; Chaudhari, M.; Pathak, N. R..2024.Effect of yoga on pulse rate and blood pressure | Wrong study design |
| 150 | Poyil, S. K.; Sekar, G.; Murugesan, S.; Parvathy, S..2024.Effect of Yogic Practices on Systolic Blood Pressure and Body Mass Index among Stressed Middle-aged Police Personnel in Kerala Police Academy | Wrong study design |
| 151 | Shrimal, P. J.; Maharana, S.; Dave, A.; Metri, K. G.; Raghuram, N.; Shrimal, S..2024.Impact of Yoga on anxiety, stress and sleep quality among health care professionals during a public health crisis | Wrong study design |

2026

| 1 | Sujan 2025 Yoga-based breathing and relaxation as adjunctive therapy for chronic migraine: A randomized controlled trial on clinical outcomes and autonomic regulation 10.1016/j.ctim.2025.103291 | Exclusion reason: Wrong population |
| --- | --- | --- |
| 2 | Chauhan 2025 An exploratory study on the changes in immune and metabolic parameters by 10 weeks of yoga intervention among medical students 10.1038/s41598-025-22421-4 | Exclusion reason: Wrong population; |
| 3 | Rathinam 2025 Effects of a 12-Week Yoga and Mindfulness Meditation Program on Psychological Stress, Inflammatory Markers, and Sleep Quality in Middle-Aged Obese Women: A Randomized Controlled Trial 10.26773/smj.251010 | Exclusion reason: Wrong comparator |
| 4 | Joss 2025 PCC-hippocampal functional connectivity associated with stress biomarker changes after meditation training for healthy adults 10.1016/j.neulet.2025.138272 | Exclusion reason: Wrong population |
| 5 | Basu 2025 The Role of Yoga in Mitigating Pulmonary Function and Psychological Decline Due to Air Pollution: A Randomized Controlled Trial in Delhi-NCR | Exclusion reason: Wrong population |
| 6 | SenthilKumaran 2025 IMPACT OF YOGIC AND PLYOMETRIC TRAINING ON SELECTED PHYSICAL VARIABLES AMONG COLLEGE MEN CRICKET PLAYERS | Exclusion reason: Wrong outcome |
| 7 | Rahaman 2025 Investigating the Role of Yogic Practices in Enhancing Respiratory and Cardiovascular Function: An Intervention Study 10.15391/prrht.2025-10(3).09 | Exclusion reason: Wrong population; |
| 8 | Saha 2025 Twelve-Week MSRT Effects on Heart Rate, Blood Pressure and Sleep Quality in Injured National Athletes 10.15391/prrht.2025-10(3).08 | Exclusion reason: Wrong intervention |
| 9 | Bhasin 2025 Therapeutic Yoga: A feasible complementary approach for glycemic control in individuals with impaired fasting glucose and elevated HbA1c 10.1016/j.conctc.2025.101493 | Exclusion reason: Wrong study design; |
| 10 | Ghosh 2025 Analyzing the Impact of Integrated Yoga and Neuro-Linguistic Programming on Blood Pressure, Stress, Anxiety, and Parenting Sense of Competence in Mothers of Adolescents: A Randomized Controlled Trial 10.17309/tmfv.2025.3.01 | Exclusion reason: Wrong population; |
| 11 | Momeni 2025 Cyclic yoga improves anthropometric indices, musculoskeletal disorders, and blood pressure in middle-aged women 10.1016/j.jbmt.2024.11.020 | Exclusion reason: Wrong population; |
| 12 | Saroja 2025 Impact of Varied Integrated Modules of Yogic Practices on Selected Physiological Variable Among Middle Aged Women 10.52783/jns.v14.1705 | Exclusion reason: Wrong population; |
| 13 | Johnson 2025 Online Yoga Pilot Intervention for Black Women at High Cardiovascular Risk: Internet-Based Recruitment and Engagement 10.2196/41221 | Exclusion reason: Wrong study design; |
| 14 | Khuntia 2025 Enhancing Athletic Performance Through Yoga: A Comparative Analysis Of University Rugby, Football, And Volleyball Players 10.53555/jab.v11i4.385 | Exclusion reason: Wrong study design |
| 15 | Ravindra 2025 A Study On The Role Of Yoga In Managing Obesity: A Therapeutic Lifestyle Approach 10.53555/jab.v11i3.297 | Exclusion reason: Wrong outcome |
| 16 | Rahaman 2025 Yogic Practices for Modulating Hematological Indices and Inflammatory Markers: A Non-Pharmacological Approach 10.7575/aiac.ijkss.v.13n.2p.101 | Exclusion reason: Wrong population |
| 17 | Tripathi 2025 Does yoga change the psycho-physiological states among first year college students? A novel investigation via yoga for stress management protocol 10.1080/02673843.2025.2502559 | Exclusion reason: Wrong population |
| 18 | Supriya 2025 Exploring the Effects of a Six-Month Restorative Yoga Trial on Metabolic Syndrome Risk Factors and Mental Health in Hong Kong Adults: The Yoga Education Series‚ÄîHong Kong 1 (YES-HK1) Feasibility Study 10.5334/paah.414 | Exclusion reason: Wrong population |
| 19 | Khodnapur 2024 Yoga Improves Vascular stiffness in COVID-19 Survivors of Vijayapur, Karnataka, India 10.13005/bpj/3038 | Exclusion reason: Wrong study design |
| 20 | Naveen 2024 Effect of tele-yoga on burnout, mental health and immune markers of health care workers on COVID-19 duty: An open-label parallel group pilot randomized controlled trial 10.1016/j.ctim.2024.103109 | Exclusion reason: Wrong population |
| 21 | Archana 2024 Effect of a Yogic Breathing Technique on Immune Parameters among Healthcare Workers: A Randomized Controlled Pilot Study 10.18502/tim.v9i4.17474 | Exclusion reason: Wrong intervention |
| 22 | Solovyeva 2024 The Impact of Exercise, Diet, and Meditation on Cognitive Function, Prefrontal Hemodynamics, Functional Connectivity, and Biochemical Parameters 10.15540/nr.11.4.355 | Exclusion reason: Wrong intervention |
| 23 | Saboo 2024 The Effect of a Yoga Lifestyle on QRISK3 Score Among Individuals at High Risk for Cardiovascular Disease 10.17761/2024-D-23-00055 | Exclusion reason: Report not retrieved |
| 24 | Naduvanthody 2025 Efficacy of Yogic Practices on Quality of Sleep, Stress, and Blood Pressure Among Shift-Based Working Security Personnel. | Exclusion reason: Report not retrieved |
| 25 | Malhotra 2025 Integrating Kriya Yoga, Pranayama and Brainwave Entrainment for Stress Reduction: An HRV-Based Exploration. | Exclusion reason: Wrong study design |
| 26 | Ghosh 2025 Effect of 21 days of yogic exercises (Suryanamaskar) on orthostatic and neurovestibular responses following 4 hours of head-down tilt https://dx.doi.org/10.25259/IJASM_8_2023 | Exclusion reason: Wrong study design |
| 27 | Govindaraj 2025 Cardio Diabetic Benefits of Yogic Approach https://dx.doi.org/10.1007/s13410-025-01585-9 | Exclusion reason: Report not retrieved |
| 28 | Nandal 2025 Effect of Anulom Vilom and Savitri Pranayama on Cardiovascular Parameters and Sleep in Medical Students https://dx.doi.org/10.69605/ijlbpr_14.8.2025.168 | Exclusion reason: Wrong intervention |
| 29 | Dahiya 2025 Impact of Makarasana on Cardiovascular Parameters: A Prospective Interventional Study https://dx.doi.org/10.7860/JCDR/2025/77677.21370 | Exclusion reason: Wrong population |
| 30 | Balaraja 2025 Yoga is non inferior to exercise in improving hepatic steatosis in MASLD - An open label, non-inferiority, randomised controlled trial https://dx.doi.org/10.1016/j.jceh.2025.102640 | Exclusion reason: Report not retrieved |
| 31 | Bhide 2025 Effectiveness of integrated yoga as a wellness strategy for a positive impact on psychological and physiological parameters in healthy healthcare students: An interventional study https://dx.doi.org/10.51248/v45i2.81 | Exclusion reason: Wrong population |
| 32 | Yellanki 2025 Role of One Month Yoga Training on Arterial Stiffness in Young Adults with Familial Hypertension: A Prospective Interventional Study https://dx.doi.org/10.7860/JCDR/2025/76346.21182 | Exclusion reason: Wrong population |
| 33 | Khandelwal 2025 Five Weeks to Better Autonomic Function: Insights from Yogic Breathing https://dx.doi.org/10.5083/ejcm/25-05-89 | Exclusion reason: Wrong study design |
| 34 | Bhanderi 2025 Effect of Yoga on Mental Stress, Lipid Profile, and BMI in Wives of BSF Personnel https://dx.doi.org/10.4103/jpbs.jpbs_327_25 | Exclusion reason: Wrong study design; |
| 35 | Sharma 2024 Evaluate the Impact of Yoga Practice on the Cardiovascular System in Healthy Individuals: A Comparative Study | Exclusion reason: Wrong study design; |
| 36 | Gupt 2025 THE OUTCOME OF SURYA NAMASKAR ON AUTONOMIC NERVOUS SYSTEM REGULATION: A COMPREHENSIVE STUDY https://dx.doi.org/10.70034/ijmedph.2025.1.283 | Exclusion reason: Wrong study design |
| 37 | Yadav 2021 EFFECT OF A 12-WEEK YOGA-BASED LIFESTYLE INTERVENTION ON VASCULAR INFLAMMATORY MARKERS IN PRE-DIABETIC OVERWEIGHT/OBESE INDIAN ADULTS | Exclusion reason: Report not retrieved |
| 38 | Pavithra 2024 Effect of Yogic Asanas on Autonomic Functions Tests in Premenstrual Syndrome Medical Students | Exclusion reason: Wrong population |
| 39 | Kumar 2024 A Study on the Impact of Yoga on Cardiovascular Workload in Prehypertensive Patients in Darbhanga Medical College Staff and Students | Exclusion reason: Wrong population; |
| 40 | Koch 2022 Effects of a yoga-based stress intervention program on the blood pressure of young police officers: A randomized controlled trial. https://dx.doi.org/10.1089/jicm.2021.0294 | Exclusion reason: Wrong population |
| 41 | Kumari 2024 Effects of Yogic Practices Synchronized With Bandha and Kumbhaka on Biological and Psychological Factors of Aging in COVID-19-Recovered Patients: a Randomized Controlled Trial 10.7759/cureus.71884 | Exclusion reason: Wrong population |
| 42 | Lavretsky 2023 Yoga for prevention of cognitive decline in older women with cardiovascular risk factors and cognitive decline 10.1002/alz.073853 | Exclusion reason: Report not retrieved |
| 43 | Valappil 2025 Optimizing the potentials of field hockey players through complex and contrast training on physiological and biochemical responses. 10.55860/n199ef76 | Exclusion reason: Wrong population |
| 44 | Rahaman 2025 Yogic Practices and Their Influence on Hematological Responses: A Physiological Perspective. 10.17309/tmfv.2025.5.22 | Exclusion reason: Wrong population |
| 45 | Mitra 2025 Yoga for Psychophysiological Wellbeing during Menstrual Phases in Eumenorrheic Females. 10.1007/s10484-024-09678-7 | Exclusion reason: Wrong population |
| 46 | Choudhary 2025 The Impact of a 12-Week Structured Yoga Intervention on Flexibility, Balance, and Joint Kinematics in University Athletes: A Quasi-Experimental Study. 10.17309/tmfv.2025.4.22 | Exclusion reason: Wrong population |
| 47 | Hidayat 2025 Enhancing physical fitness in older adults: a six-month medium intensity training intervention yields significant improvements. 10.1186/s13102-025-01401-1 | Exclusion reason: Wrong intervention |
| 48 | Kumar 2025 Evaluating the Effect of Yogic Intervention on Psychological Distress Among Injured Athletes. 10.17309/tmfv.2025.4.06 | Exclusion reason: Wrong population |
| 49 | Badve 2025 Considering the Impact of Yoga on Psychological Well-being and Quality of Life in Perimenopausal Women: A Randomized Controlled Trial. 10.17309/tmfv.2025.2.14 | Exclusion reason: Wrong outcome |
| 50 | Palanao 2025 Effects of yoga exercise combined with passive stretching on the flexibility and strength of female students. 10.47197/re-tos.v69.116707 | Exclusion reason: Wrong population; |
| 51 | DEMƒ∞RLƒ∞ 2025 Aerial Yoga ve Reformer Pilatesin Core Stabilizasyonu, Esneklik ve Denge √úzerindeki Etkileri: Sedanter Kadƒ±nlar √úzerine Bir Kar≈üƒ±la≈ütƒ±rma. | Exclusion reason: Wrong population |
| 52 | Nontakhod 2024 Effects of yoga exercise on heart rate in post COVID-19 patients. 10.47197/retos.v61.108844 | Exclusion reason: Wrong population |
| 53 | Pramanik 2025 Yogic Practices as a Complementary Approach to Physical Fitness: An Intervention Study. 10.17309/tmfv.2025.1.14 | Exclusion reason: Wrong population |
| 54 | L√≥pez-Fuenzalida 2025 Effects of a Kundalini Yoga programme on cardiorespiratory fitness and health-related quality of life in university students. 10.47197/retos.v63.110 | Exclusion reason: Wrong outcome; |
| 55 | BALOGH 2025 Comparative analysis of ten-week high-intensity interval training, moderate-intensity continuous training, and proprioceptive workouts: Impact on cognitive abilities, body composition, perceived stress and motor skills. 10.7752/jpes.2025.03050 | Exclusion reason: Wrong population |
| 56 | Manaswin 2025 COMPARATIVE EFFECTS OF SURYANAMASKAR AND DYNAMIC STRETCHING ON THE FLEXIBILITY OF CRICKET PLAYERS. | Exclusion reason: Wrong population |
| 57 | Oz 2024 Yoga, Physical Therapy and Home Exercise Effects on Chronic Low Back Pain: Pain Perception, Function, Stress, and Quality of Life in a Randomized Trial. 10.1177/00315125241292235 | Exclusion reason: Wrong outcome |
| 58 | Saboo 2024 A Study on Yoga-Based Lifestyle Intervention versus Dietary Intervention Alone on Cardiometabolic Risk Factors among People with Prediabetes 10.4103/aam.aam_56_23 | Exclusion reason: Wrong population; |
| 59 | Shelke 2024 Impact of Yoga Practice on Lipid Profile and Insulin Resistance in Overweight and Obese Individuals - A Pilot Study 10.4103/jss.jss_346_23 | Exclusion reason: Wrong study design; |
| 60 | Gamboa 2025 Blood Pressure and Autonomic Changes From 12-Weeks of Yoga-Based Slow Breathing Exercises 10.1177/27536130251380265 | Exclusion reason: Wrong intervention; |
| 61 | Szaszk√≥ 2024 Testing the impact of hatha yoga on task switching: a randomized controlled trial 10.3389/fnhum.2024.1438017 | Exclusion reason: Wrong population |
| 62 | Sharma 2025 A Study to Evaluate Role of Integrative Yoga-based Interventions on Cognitive and Autonomic Functions among Major Depressive Disorder 10.4103/ijoy.ijoy_194_24 | Exclusion reason: Wrong study design |
| 63 | Anjali 2025 A Comparative Clinical Study to Evaluate The Efficacy of Lekhana Basti in Chaturbhadra Kalpa Basti Krama and Yoga Basti Krama in the Management of Sthoulya w.s.r to Obesity 10.47552/ijam.v16i2.5323 | Exclusion reason: Wrong intervention; |
