# Supplementary material for: Impact of yoga on cardiometabolic health in adults with overweight or obesity: A systematic review and meta-analysis of randomized controlled trials
Source: PLOS Glob Public Health. 2026 Apr 22;6(4):e0006174. doi: 10.1371/journal.pgph.0006174 (PMC13102220; doi:10.1371/journal.pgph.0006174)
Supplement: S2 Table — (DOCX) [file pgph.0006174.s004.docx]

| **No** | **Author, Year** | **Sample characteristics** | | | | | **Characteristics of intervention** | | | | | | |
| --- | --- | --- | --- | --- | --- | --- | --- | --- | --- | --- | --- | --- | --- |
|  |  | **n** | **Proportion of male** | **BMI**  **(Mean** ±  **SD)** | **Ethnicity** | **Health condition** | **Type of yoga** | **Components of yoga** | **Frequency (days /week)** | **Duration of session (minutes)** | **Duration of intervention (weeks)** | **Supervision** | **Comparator** |
| 1 | Chauhan 2017 | Yoga group: 64 Control group: 26 | 43% male | Yoga group: 26.4 ± 2.5 Control group: 26.40 ± 4.36 | Asian (India) | High SBP; High DBP | NI | Postures, breathing, and meditation | 7 | 60 | 4 | Supervised | Inactive Control |
| 2 | Cramer 2016 | Yoga group: 40 Control group: 20 | 0% male | Yoga group: 32.9±4.1  Control group: 36.9±6.6 | Non-asian (Germany) | High SBP; High DBP | Traditional hatha yoga | Postures, breathing, and meditation | 2 | 80-100 | 12 | Supervised + Practice at home | Inactive control |
| 3 | Hegde 2013 | Yoga group: 14  Control group: 15 | 48% Male | Yoga group: 27.2 ± 3.7; Control group: 26.2 ± 4.3 | Asian (India) | High SBP; High DBP; High HbA1c; | NI | Postures, breathing, and meditation | 5 | 75-90 | 12 | Supervised | Inactive control |
| 4 | Hewett 2017 | Yoga group: 29 Control group: 34 | 21% male | Yoga group: 29.9 ± 6.2 Control group: 30.9 ± 6.3 | Non-asian (Australia) | High SBP | Bikram yoga | Postures, breathing, and meditation | 3-5 | 90 | 16 | Supervised | Inactive Control |
| 5 | Hunter 2018 | Yoga group: 14  Control group: 19 | 27% male | Yoga group: 29 ± 4  Control group: 31 ± 7 | Non-asian (USA) | High SBP; High TC; High TG | Bikram Yoga | Postures and breathing | 3 | 90 | 12 | Supervised | Inactive control |
| 6 | Jabir 2017 | Yoga group: 50  Control group: 50 | 50% male | Yoga group: 23.48±4.31; Control group: 24.31±2.91 | Asian (India) | High SBP; High DBP | Balasana training | Posture and breathing | NI | NI | 12 | Supervised | Inactive control |
| 7 | Kaur 2021 | Yoga group: 91  Control group: 93 | 26% male | Yoga group: 28.59±5.75; Control group: 28.53±5.01 | Asian (India) | Not desirable HDL | Diabetic yoga protocol (DYP) | Postures, breathing, and meditation | NI | 60 | 12 | Supervised | Inactive control |
| 8 | Keerthi, 2017 | Yoga group: 61 Control group: 60 | 53,8% male | All: 27.15±3.90 | Asian (India) | High FPG | NI | Postures, breathing, and meditation | 3 | 45 | 12 | Supervised + Practice at home | Inactive control |
| 9 | Kim 2012 | Yoga group: 16  Control group: 18 | 0% male | Yoga group: 26.0 (1.0); Control group: 27.0 (1.0) | Non-asian (USA) | High SBP | Ashtanga Yoga | Postures and meditation | 2 | 60 | 32 | Supervised | Inactive control |
| 10 | Lee 2012 | Yoga group: 8  Control group: 8 | 0% male | Yoga group: 25.19 ± 1.71 Control group: 25.13 ± 1.63 | Asian (Korea) | High TC, High LDL, High SBP, High DBP  High FBG | NI | Postures, and breathing | 3 | 60 | 16 | Supervised | Inactive control |
| 11 | Mandal 2021 | Yoga group: 19 Control group: 32 | 28% male | Yoga group: 25.9 + 4.2; Control group: 24.4 + 4.4 | Asian (India) | Healthy | NI | Postures, breathing, and meditation | 2 | 50 | 12 | Supervised | Inactive control |
| 12 | Pal 2015 | Yoga group: 34 Control group: 30 | 100% male | Yoga group: 25.7 ± 0.43; Control group: 27.4 ± 1.43 | Asian (India) | Healthy | NI | Postures, breathing, and meditation | 6 | 60 | 12 | Supervised | Inactive control |
| 13 | Patil, 2019 | Yoga group: 28 Control group: 29 | NI | Yoga group: 23.19±2.52; Control group: 23.56±3.36 | Asian (India) | High SBP | NI | Postures, breathing, and meditation | 5 | 60 | 8 | Supervised | Inactive control |
| 14 | Thiyagarajan 2015 | Yoga group: 51  Control group: 49 | 62% male | Yoga group: 25.74±3.52; Control group: 25.71±3.21 | Asian (India) | High SBP, High DBP, Not desirable HDL | Yoga therapy protocol for hypertension designed  in ACYTER, JIPMER | Postures, breathing, and meditation | 3 | 45 | 2 | Supervised + Practice at home | Inactive control |
| 15 | Wahyuni 2021 | Yoga group: 18  Control group: 19 | NI | NI | Asian (Indonesia) | Healthy | NI | Postures, breathing, and meditation | 2 | 60 | 8 | Supervised | Inactive control |
| 16 | Yang 2011 | Yoga group: 12 Control group: 10 | 9% male | Yoga group: 28.2±3.7  Control group: 31.5±6.2 | Non-asian (USA) | High TC | Vinyasa style yoga (evolving form of hatha yoga) | Postures, and breathing | 2 | 60 | 12 | Supervised + Practice at home | Inactive control |
| 17 | Poojari 2024 | Yoga group: 14  Control group: 15 | 38% male | Yoga group: 25.8  Control group: 23.8 | Asian (India) | No specific information on blood pressure, blood glucose, and lipid profile | The integrated approach of yoga therapy (IAYT) | Postures, breathing, and meditation | 6 | 45 | 12 | Supervised + Practice at home | Inactive control |
| 18 | Kumar 2024 | Yoga group: 50  Control group: 50 | NI | Yoga group: 24.5 ± 2.3  Control group: No information | Asian (India) | Healthy | NI | Postures, breathing, and meditation | 5 | 30 | 24 | Supervised | Inactive control |
| 19 | Karlekar 2024 | Yoga group: 28  Control group: 28 | 0% male | Yoga group: 26.11 ± 8.89 Control group: 24.89 ± 4.78 | Asian (India) | High SBP | NI | Postures, breathing, and meditation | 7 | 60 | 12 | Supervised | Inactive control |
| 20 | Sharma 2024 | Yoga group: 40  Control group: 46 | 16% male | Yoga group: 26.15 ± 4.20  Control group: 26.78 ±5.14 | Asian (India) | High SBP | Raja Yoga | Postures, breathing, and meditation | 3 | NI | 8 | Supervised | Inactive control |
| 21 | Madhu 2024 | Yoga group: 391  Control group:383 | 42% male | Yoga group: 28.9 ± 4.3  Control group: 28.7 ± 4.7 | Asian (India) | High SBP Prediabetes Not desirable HDL | NI | Posture and breathing | 5 | 40 | 144 | Supervised + practice at home | Inactive control |
| 22 | Mitra 2023 | Yoga group: 37  Control group: 37 | 0% male | Yoga group: 23.10 ± 0.33  Control group: 22.68 ± 0.54 | Asian (India) | High SBP | NI | Postures, breathing, and meditation | 5 | 40 | 12 | Supervised | Inactive control |
| 23 | Mitra 2024 | Yoga group: 30 Control group: 30 | 0% male | Yoga group: 23.49 ± 2.79  Control group: 25.2 ± 3.15 | Asian (India) | High SBP | NI | Postures, breathing, and meditation | 5 | 60 | 12 | Supervised | Inactive control |
| 24 | Yamuna 2024 | Yoga group: 43  Control group: 45 | 100% male | Yoga group: 25.98 – 3.91  Control group: 26.38 – 10.43 | Asian (India) | Healthy | NI | Postures, breathing, and meditation | 6 | 60 | 12 | Supervised | Inactive control |
| 25 | Sharma 2023 | Yoga group: 42  Control group: 46 | 15% male | Yoga group: 26.08±4.70  Control group: 26.78±5.14 | Asian (India) | High SBP  Not desirable HDL | Standard common yoga protocol | Postures, breathing, and meditation | 3 | 40 | 8 | Supervised | Inactive control |
| 26 | McDermott, 2014 | Yoga group: 21  Control group: 20 | 39% male | Yoga group: 28.4±5.3; Control group: 26.9±3.0 | Asian (India) | High FBG, High PPBG, High SBP, High DBP, High TC, High TG | NI | Posture and breathing | 3-6 | 75 | 8 | Supervised + Practice at home | Other exercises (Walking) |
| 27 | Kurian 2023 | Yoga group: 19  Control group: 20 | 28% male | Yoga group: 26.87±2.68  Control group: 27.0315789±1.828950103 | Asian (India) | Prediabetes  Not desirable HDL | NI | Postures, breathing, and meditation | 5 | 45 | 12 | Supervised | walking |
| 28 | Rajbhoj 2023 | Yoga group: 22  Control group: 15 | 100% male | Yoga group: 23.04 ± 3.89  Control group: 21.21±4.50 | Asian (India) | Healthy | NI | Postures, breathing, and meditation | 6 | NI | 16 | Supervised | inactive control |
| 29 | Lu 2025 | Yoga group: 8 Control group: 10 | 77.8% male | Yoga group: 28.97 ± 3.13  Control group: 23.65 ± 1.71 | Non-Asian (USA) | High SBP | NI | Postures, breathing, and meditation | 2 | 90 | 6 | Supervised | Moderate intensity aerobic exercise |
| 30 | Denninger 2025 | Yoga group: 68 Control group: 70 | 37% male | Yoga group: 25.20 Control group:  25.20 | Non-Asian (USA) | Healthy | Kundalini Yoga | Postures, breathing, and meditation | 1 | 45 | 8 | Supervised + Practice at home | Inactive control |
