# Supplementary material for: Impact of yoga on cardiometabolic health in adults with overweight or obesity: A systematic review and meta-analysis of randomized controlled trials
Source: PLOS Glob Public Health. 2026 Apr 22;6(4):e0006174. doi: 10.1371/journal.pgph.0006174 (PMC13102220; doi:10.1371/journal.pgph.0006174)
Supplement: S3 Table — (DOCX) [file pgph.0006174.s005.docx]

Vote counting on blood pressure, lipid profile, glucose metabolism, redox and inflammation outcomes

| **No** | **Author, Year** |  |  | | |  |  | | **Results** | | | | | | | | | | | | | | | | | | | | | | | |  |
| --- | --- | --- | --- | --- | --- | --- | --- | --- | --- | --- | --- | --- | --- | --- | --- | --- | --- | --- | --- | --- | --- | --- | --- | --- | --- | --- | --- | --- | --- | --- | --- | --- | --- |
|  |  | **Blood pressure** | | | **Lipid profile** | | | | | | | | **Glucose parameters** | | | | **Pro-oxidant markers** | | **Antioxidant markers** | | | | | **Pro-inflammatory markers** | | | | | | | **Anti-inflammatory markers** | | |
|  |  | **SBP** | | **DBP** | **LDL** | | | **HDL** | | **VLDL** | **TG** | **TC** | **FBG** | **PPBG** | **HbA1C** | **HOMA-IR** | **MDA** | **Hcy** | **GSH** | **Vit C** | **Vit E** | **SOD/**  **MnSOD** | **Catalase** | **hsCRP** | **TNF Alpha** | **IL-1** | **IL-6** | **sIL-2R** | **IL-10** | | | **Adiponectin** | |
|  | Compared to inactive control |  | |  |  | | |  | |  |  |  |  |  |  |  |  |  |  |  |  |  |  |  |  |  |  |  |  | | |  | |
| 1 | Chauhan 2017^55^ | ▼ | | ▼ | NI | | | NI | | NI | NI | NI | NI | NI | NI | NI | NI | NI | NI | NI | NI | NI | NI | NI | NI | NI | NI | NI | NI | | | NI | |
| 2 | Cramer 2016^56^ | ▽ | | △ | NI | | | NI | | NI | NI | NI | NI | NI | NI | NI | NI | NI | NI | NI | NI | NI | NI | NI | NI | NI | NI | NI | NI | | | NI | |
| 3 | Hegde 2013^57^ | ▼ | | ▽ | NI | | | NI | | NI | NI | NI | ▼ | ▽ | ▽ | NI | ▼ | NI | △ | △ | ▽ | ▼ | NI | NI | NI | NI | NI | NI | NI | | | NI | |
| 4 | Hewett 2017^58^ | △ | | △ | ▽ | | | ▽ | | NI | △ | ▽ | NI | NI | NI | NI | NI | NI | NI | NI | NI | NI | NI | △ | NI | NI | NI | NI | NI | | | NI | |
| 5 | Hunter 2018^59^ | △ | | △ | △ | | | ▽ | | NI | △ | ▭ | ▭ | NI | NI | NI | NI | NI | NI | NI | NI | NI | NI | NI | NI | NI | NI | NI | NI | | | NI | |
| 6 | Jabir 2017^60^ | ▼ | | ▼ | NI | | | NI | | NI | NI | NI | NI | NI | NI | NI | NI | NI | NI | NI | NI | NI | NI | NI | NI | NI | NI | NI | NI | | | NI | |
| 7 | Kaur 2021^61^ | NI | | NI | ▼ | | | △ | | ▽ | ▽ | ▽ | △ | ▼ | ▽ | NI | NI | NI | NI | NI | NI | NI | NI | NI | NI | NI | NI | NI | NI | | | NI | |
| 8 | Keerthi, 2017^62^ | NI | | NI | NI | | | NI | | NI | NI | NI | ▼ | NI | NI | ▼ | NI | NI | NI | NI | NI | NI | NI | NI | NI | NI | NI | NI | NI | | | NI | |
| 9 | Kim 2012^63^ | ▽ | | ▽ | NI | | | NI | | NI | NI | NI | NI | NI | NI | NI | NI | NI | NI | NI | NI | NI | NI | NI | NI | NI | NI | NI | NI | | | NI | |
| 10 | Lee 2012^64^ | ▼ | | ▼ | ▼ | | | ▲ | | NI | ▼ | ▼ | ▼ | NI | NI | ▼ | NI | NI | NI | NI | NI | NI | NI | NI | NI | NI | NI | NI | NI | | | ▲ | |
| 11 | Mandal 2021^65^ | ▼ | | ▽ | NI | | | NI | | NI | NI | NI | NI | NI | NI | NI | NI | NI | NI | NI | NI | NI | NI | ▽ | NI | NI | NI | NI | NI | | | NI | |
| 12 | Pal 2015^66^ | NI | | NI | NI | | | NI | | NI | NI | NI | NI | NI | NI | NI | ▽ | NI | ▲ | ▲ | ▲ | ▲ | NI | NI | NI | NI | NI | NI | NI | | | NI | |
| 13 | Patil, 2019^67^ | NI | | NI | NI | | | NI | | NI | NI | NI | ▼ | ▼ | NI | ▼ | NI | NI | NI | NI | NI | NI | NI | NI | NI | NI | NI | NI | NI | | | NI | |
| 14 | Thiyagarajan 2015^68^ | ▼ | | ▽ | ▼ | | | ▲ | | NI | ▼ | ▼ | ▼ | NI | NI | NI | NI | NI | NI | NI | NI | NI | NI | NI | NI | NI | NI | NI | NI | | | NI | |
| 15 | Wahyuni 2021^69^ | NI | | NI | NI | | | NI | | NI | NI | NI | NI | NI | NI | NI | NI | NI | NI | NI | NI | ▲ | NI | NI | NI | NI | NI | NI | NI | | | NI | |
| 16 | Yang 2011^70^ | ▽ | | ▽ | ▽ | | | △ | | NI | ▽ | ▽ | ▽ | NI | NI | NI | NI | NI | NI | NI | NI | NI | NI | NI | NI | NI | NI | NI | NI | | | NI | |
| 17 | Poojari 2024^71^ | NI | | NI | NI | | | NI | | NI | NI | NI | NI | NI | NI | NI | NI | NI | NI | NI | NI | NI | NI | NI | ▼ | NI | NI | NI | NI | | | NI | |
| 18 | Kumar 2024^72^ | ▼ | | NI | NI | | | NI | | NI | NI | NI | NI | NI | NI | NI | NI | NI | NI | NI | NI | NI | NI | NI | NI | NI | NI | NI | NI | | | NI | |
| 19 | Karlekar 2024^73^ | ▼ | | ▼ | NI | | | NI | | NI | NI | NI | NI | NI | NI | NI | NI | NI | NI | NI | NI | NI | NI | NI | NI | NI | NI | NI | NI | | | NI | |
| 20 | Sharma 2024^74^ | ▼ | | ▼ | NI | | | NI | | NI | NI | NI | NI | NI | NI | NI | NI | NI | NI | NI | NI | NI | NI | ▽ | ▼ | ▼ | ▼ | NI | △ | | | NI | |
| 21 | Madhu 2024^75^ | NI | | NI | △ | | | ▼ | | NI | ▽ | △ | △ | △ | ▲ | ▽ | NI | NI | NI | NI | NI | NI | NI | NI | NI | NI | NI | NI | NI | | | NI | |
| 22 | Mitra 2023^76^ | ▼ | | ▽ | ▽ | | | ▲ | | NI | ▼ | NI | NI | NI | NI | NI | NI | NI | ▲ | NI | NI | ▲ | ▲ | NI | ▼ | NI | NI | NI | ▲ | | | NI | |
| 23 | Mitra 2024^77^ | ▼ | | ▽ | NI | | | NI | | NI | NI | NI | NI | NI | NI | NI | NI | NI | NI | NI | NI | NI | NI | NI | NI | NI | NI | NI | NI | | | NI | |
| 24 | Yamuna 2024^78^ | NI | | NI | NI | | | NI | | NI | NI | NI | NI | NI | NI | NI | NI | ▼ | NI | NI | NI | NI | NI | NI | NI | NI | NI | NI | NI | | | NI | |
| 25 | Sharma 2023^79^ | ▼ | | ▼ | ▼ | | | ▲ | | ▼ | ▼ | ▼ | NI | NI | NI | NI | NI | NI | NI | NI | NI | NI | NI | ▽ | ▼ | ▼ | ▼ | NI | △ | | | NI | |
| 25 | Rajbhoj 2023^80^ | NI | | NI | NI | | | NI | | NI | NI | NI | NI | NI | NI | NI | NI | NI | NI | NI | NI | NI | NI | NI | NI | NI | NI | ▼ | NI | | | NI | |
|  | Compared to other exercises |  | |  |  | | |  | |  |  |  |  |  |  |  |  |  |  |  |  |  |  |  |  | | | | |  | | |  |
| 27 | McDermott, 2014^81^ | △ | | ▽ | ▽ | | | NI | | NI | ▭ | ▽ | △ | ▽ | NI | NI | NI | NI | NI | NI | NI | NI | NI | NI | NI | NI | NI | NI | NI | | | NI | |
| 28 | Kurian 2023^82^ | NI | | NI | ▼ | | | ▲ | | ▽ | ▼ | NI | ▼ | NI | NI | ▼ | NI | NI | NI | NI | NI | NI | NI | NI | NI | NI | NI | NI | NI | | | NI | |
| 29 | Lu 2025^90^ | ▽ | | ▽ | NI | | | NI | | NI | NI | NI | NI | NI | NI | NI | NI | NI | NI | NI | NI | NI | NI | NI | NI | NI | NI | NI | NI | | | NI | |
| 30 | Denninger 2025^91^ | ▽ | | ▽ | NI | | | △ | | NI | NI | △ | NI | NI | △ | NI | NI | NI | NI | NI | NI | NI | NI | NI | NI | NI | △ | NI | NI | | | NI | |

NI: No information; BMI: Body mass index; SBP: Systolic blood pressure; DBP: Diastolic blood pressure; LDL: Low Density Lipoprotein; HDL: High Density Lipoprotein; VLDL: Very Low-Density Lipoprotein; TG: Triglycerides; TC: Total cholesterol; FBG: Fasting blood glucose; PPBG: Post prandial blood glucose; HbA1c: Hemoglobin A1c; HOMA-IR: Homeostatic Model Assessment of Insulin Resistance; Hcy: Homocysteine; GSH: Glutathione; SOD: Superoxide dismutase; MDA: Malondialdehyde; hsCRP: High-sensitivity C-reactive protein; IL: interleukin; sIL-2R: soluble interleukin-2 receptor; ▲: statistically significant increased; ▼: statistically significant decreased; △: non-statistically significant increased; ▽: non-statistically significant decreased; ▭: non-statistically significant and no specific direction;.
