# Supplementary material for: Impact of yoga on cardiometabolic health in adults with overweight or obesity: A systematic review and meta-analysis of randomized controlled trials
Source: PLOS Glob Public Health. 2026 Apr 22;6(4):e0006174. doi: 10.1371/journal.pgph.0006174 (PMC13102220; doi:10.1371/journal.pgph.0006174)
Supplement: S4 Table — (DOCX) [file pgph.0006174.s006.docx]

| Study | D1 | D2 | D3 | D4 | D5 | Overall |
| --- | --- | --- | --- | --- | --- | --- |
| Chauhan 2017 | High | Not assessed | Low | Low | Some concerns | High |
| Cramer 2016 | Low | Not assessed | Low | Low | Some concerns | Some concerns |
| Hedge 2013 | Low | Not assessed | Low | Low | Some concerns | Some concerns |
| Hewett 2017 | Low | Not assessed | Low | Low | Low | Low |
| Hunter 2018 | Low | Not assessed | Low | Low | Some concerns | Some concerns |
| Jabir 2017 | Some concerns | Not assessed | Low | High | Some concerns | High |
| Kaur 2021 | Low | Not assessed | Low | Low | Low | Low |
| Keerthi 2017 | Low | Not assessed | Low | Low | Some concerns | Some concerns |
| Kim 2012 | Some concerns | Not assessed | Low | Low | Some concerns | Some concerns |
| Lee 2012 | Some concerns | Not assessed | Low | Low | Some concerns | Some concerns |
| Mandal 2021 | Some concerns | Not assessed | High | Low | Some concerns | High |
| Pal 2015 | Some concerns | Not assessed | Low | Low | Some concerns | Some concerns |
| Patil 2019 | Some concerns | Not assessed | Low | Low | Some concerns | Some concerns |
| Thiyagarajan 2015 | Low | Not assessed | Low | Low | Some concerns | Some concerns |
| Wahyuni 2021 | Some concerns | Not assessed | Low | Low | Some concerns | Some concerns |
| Yang 2011 | Some concerns | Not assessed | Low | Low | Some concerns | Some concerns |
| Karlekar 2024 | Low | Not assessed | Low | Low | Some concerns | Some concerns |
| Madhu 2024 | Low | Not assessed | Some concerns | Low | High | High |
| Mitra 2023 | High | Not assessed | Low | High | Some concerns | High |
| Mitra 2024 | Some concerns | Not assessed | Low | High | Some concerns | High |
| Sharma 2023 | Low | Not assessed | High | Low | Low | High |
| Sharma 2024 | Low | Not assessed | High | Low | Some concerns | High |
| Ghosh 2025 | Some concerns | Not assessed | Low | Low | Low | Some concerns |
| Denninger 2025 | Some concerns | Not assessed | High | High | High | High |
