# Supplementary material for: Impact of yoga on cardiometabolic health in adults with overweight or obesity: A systematic review and meta-analysis of randomized controlled trials
Source: PLOS Glob Public Health. 2026 Apr 22;6(4):e0006174. doi: 10.1371/journal.pgph.0006174 (PMC13102220; doi:10.1371/journal.pgph.0006174)
Supplement: S5 Table — (PDF) [file pgph.0006174.s007.pdf]

Name	Effect Measure	Analysis Model	Effect Estimate	SE	CI Start	CI End	Weight	Q	P(Q)	I-Sqr(Q)	Tau-Sqr	Z	P(Z)	Qint	P(Qint)	I-Sqr(Qint)	df
Blood glucose																	
Fasting Blood Glucose	Mean Difference	Random	-0.11		-0.26	0.03	100	86.2	0	89.56	0.05	1.51	0.13	1.17	0.28	14.17	9
Asia	Mean Difference	Random	-0.15		-0.32	0.02	80.85	82.43	0	91.51	0.05	1.73	0.08				7
Non Asia	Mean Difference	Random	0.03		-0.25	0.3	19.15	2.85	0.09	64.94	0.03	0.19	0.85				1
Fasting Blood Glucose Asian without high RoB	Mean Difference	Random	-0.18		-0.4	0.03	100	82.43	0	92.72	0.07	1.64	0.1	62.08	0	95.17	6
at least 12 weeks at least 60 minutes without high RoB	Mean Difference	Random	-0.01		-0.08	0.06	45.79	0.44	0.8	0	0	0.31	0.76				2
At least 12 weeks at least 60 minutes high FBG without high RoB	Mean Difference	Random	-0.68		-0.88	-0.48	14.77	0	0	100	0	6.69	0				0
Less than 12 weeks without high RoB	Mean Difference	Random	0.32		0.17	0.47	15.56	0	1	0	0	4.05	0				0
At least 12 weeks less than 60 minutes without high RoB	Mean Difference	Random	-0.68		-1.8	0.45	23.88	14.61	0	93.16	0.62	1.18	0.24				1
At least 12 weeks, less than 60 minutes, high FBG, without high RoI	Mean Difference	Random	0		0	0	0	0	0	0	0	0	0				0
PPBG	Mean Difference	Random	-0.2		-0.62	0.23	100	72.06	0	95.84	0.17	0.91	0.36	0	1	0	3
Asia	Mean Difference	Random	-0.2		-0.62	0.23	100	72.06	0	95.84	0.17	0.91	0.36				3
Non-asia	Mean Difference	Random	0		0	0	0	0	0	0	0	0	0				0
PPBG Asia without high RoB	Mean Difference	Random	-0.14		-0.67	0.4	100	46.9	0	95.74	0.21	0.5	0.62	2.01	0.16	50.3	2
Asian at least 12 week at least 60 minutes without high RoB	Mean Difference	Random	0.02		-0.59	0.63	67.09	20.92	0	95.22	0.18	0.06	0.95				1
Asian at least 12 weeks less than 60 minutes	Mean Difference	Random	0		0	0	0	0	0	0	0	0	0				0
Asian less than 12 weeks without high RoB	Mean Difference	Random	-0.45		-0.68	-0.22	32.91	0	0	100	0	3.9	0				0
HBA1c	Mean Difference	Random	-0.04		-0.08	-0.01	100	6.55	0.04	69.46	0	2.8	0.01	0	1	0	2
Asia	Mean Difference	Random	-0.04		-0.08	-0.01	100	6.55	0.04	69.46	0	2.8	0.01				2
Non-asia	Mean Difference	Random	0		0	0	0	0	0	0	0	0	0				0
HBA1c Asia without High RoB	Mean Difference	Random	-0.05		-0.13	0.02	100	4.11	0.04	75.68	0	1.33	0.18	0	1	0	1
At least 12 weeks, at least 60 minutes without High RoB	Mean Difference	Random	-0.05		-0.13	0.02	100	4.11	0.04	75.68	0	1.33	0.18				1
At least 12 weeks, less than 60 minutes, prediabetes, without High	Mean Difference	Random	0		0	0	0	0	0	0	0	0	0				0
HOMA-IR	Mean Difference	Random	-0.87		-1.64	-0.1	100	31.29	0	93.61	0.4	2.22	0.03	0	1	0	2
Asia	Mean Difference	Random	-0.87		-1.64	-0.1	100	31.29	0	93.61	0.4	2.22	0.03				2
Non-asia	Mean Difference	Random	0		0	0	0	0	0	0	0	0	0				0
HOMA-IR Asia Without High RoB	Mean Difference	Random	-1.52		-3.62	0.57	100	20.73	0	95.18	2.18	1.42	0.15	20.73	0	95.18	1
Less than 12 weeks Without High RoB	Mean Difference	Random	-0.5		-0.7	-0.3	52.19	0	1	0	0	4.92	0				0
At least 12 weeks Without High RoB	Mean Difference	Random	-2.64		-3.54	-1.74	47.81	0	1	0	0	5.75	0				0
Lipid profile																	
VLDL	Mean Difference	Random	-0.04		-0.08	0	100	0.02	0.88	0	0	2.16	0.03	0	1	0	1
Asia	Mean Difference	Random	-0.04		-0.08	0	100	0.02	0.88	0	0	2.16	0.03				1
Non-asian	Mean Difference	Random	0		0	0	0	0	0	0	0	0	0				0
LDL	Mean Difference	Random	-0.08		-0.19	0.04	100	46.06	0	82.63	0.02	1.36	0.17	6.51	0.01	84.64	8
Asian	Mean Difference	Random	-0.14		-0.28	0	71.09	37.59	0	86.7	0.03	1.93	0.05				5
Non-asian	Mean Difference	Random	0.09		-0.02	0.2	28.91	1.17	0.56	0	0	1.67	0.09				2
LDL Asian without High RoB	Mean Difference	Random	-0.33		-0.64	-0.03	100	18.77	0	89.34	0.06	2.13	0.03	18.65	0	94.64	2
At least 12 weeks at least 60 minutes without High RoB	Mean Difference	Random	-0.48		-0.62	-0.33	64.33	0.11	0.73	0	0	6.49	0				1
At least 12 weeks less than 60 minutes without High RoB	Mean Difference	Random	-0.07		-0.19	0.05	35.67	0	1	0	0	1.19	0.24				0
Less than 12 weeks, less than 60 minutes without High RoB	Mean Difference	Random	0		0	0	0	0	0	0	0	0	0				0
LDL Non Asian	Mean Difference	Random	0.09		-0.02	0.2	100	1.17	0.56	0	0	1.67	0.09	0.55	0.46	0	2
At least 3 times/week	Mean Difference	Random	0.1		-0.01	0.21	91.35	0.62	0.43	0	0	1.82	0.07				1
Less than 2 times/week	Mean Difference	Random	-0.04		-0.4	0.32	8.65	0	1	0	0	0.22	0.83				0
HDL	Mean Difference	Random	0.06		0.01	0.12	100	32.73	0	75.55	0	2.17	0.03	0.53	0.47	0	8
Asian	Mean Difference	Random	0.08		0	0.15	81.16	32.44	0	84.59	0.01	2.09	0.04				5
Non-asian	Mean Difference	Random	0.03		-0.05	0.12	18.84	0.25	0.88	0	0	0.83	0.4				2

HDL Asian without high RoB	Mean Difference	Random	0.08	0	0.17	100	9.02	0.01	77.82	0	2	0.05	2.77	0.1	63.85	2
At least 12 weeks at least 60 minutes without high RoB	Mean Difference	Random	0.12	0.03	0.2	60.88	1.97	0.16	49.3	0	2.73	0.01				1
At least 12 weeks, less than 60 minutes without high RoB	Mean Difference	Random	0.04	-0.01	0.08	39.12	0	1	0	0	1.52	0.13				0
Less than 12 weeks, less than 60 minutes without high RoB	Mean Difference	Random	0	0	0	0	0	0	0	0	0	0				0
HDL Non Asian	Mean Difference	Random	0.03	-0.05	0.12	100	0.25	0.88	0	0	0.83	0.4	0.09	0.76	0	2
At least 3 times/week	Mean Difference	Random	0	-0.22	0.22	13.91	0.16	0.69	0	0	0.03	0.97				1
2 times/week	Mean Difference	Random	0.04	-0.05	0.13	86.09	0	1	0	0	0.88	0.38				0
Total cholesterol	Mean Difference	Random	-0.06	-0.16	0.03	100	37.59	0	81.38	0.01	1.27	0.2	2.4	0.12	58.35	7
Asian	Mean Difference	Random	-0.1	-0.22	0.02	78.61	35.62	0	88.77	0.01	1.7	0.09				4
Non-asian	Mean Difference	Random	0.06	-0.11	0.23	21.39	0.24	0.89	0	0	0.7	0.48				2
Total cholesterol Asian without high RoB	Mean Difference	Random	-0.22	-0.55	0.11	100	25.91	0	92.28	0.08	1.29	0.2	0.65	0.42	0	2
At least 12 weeks at least 60 minutes without high RoB	Mean Difference	Random	-0.31	-0.94	0.31	65.84	24	0	95.83	0.2	0.98	0.33				1
At least 12 weeks less than 60 minutes without high RoB	Mean Difference	Random	-0.05	-0.19	0.09	34.16	0	1	0	0	0.69	0.49				0
Less than 12 weeks, less than 60 minutes without high RoB	Mean Difference	Random	0	0	0	0	0	0	0	0	0	0				0
Total cholesterol Non Asian	Mean Difference	Random	0.06	-0.11	0.23	100	0.24	0.89	0	0	0.7	0.48	0.22	0.64	0	2
At least 3 times/week	Mean Difference	Random	0.04	-0.15	0.23	80.5	0.02	0.88	0	0	0.42	0.67				1
2 times/week	Mean Difference	Random	0.14	-0.24	0.52	19.5	0	1	0	0	0.73	0.47				0
Triglycerides	Mean Difference	Random	-0.26	-0.42	-0.11	100	58.83	0	86.4	0.04	3.28	0	0.05	0.82	0	8
Asian	Mean Difference	Random	-0.23	-0.4	-0.07	70.22	27.89	0	82.07	0.03	2.82	0				5
Non-asian	Mean Difference	Random	-0.3	-0.83	0.24	29.78	26.45	0	92.44	0.19	1.1	0.27				2
Triglycerides Asian without high RoB	Mean Difference	Random	-0.26	-0.46	-0.07	100	4.81	0.09	58.41	0.02	2.63	0.01	4.8	0.03	79.19	2
At least 12 weeks at least 60 minutes without high RoB	Mean Difference	Random	-0.35	-0.47	-0.23	70.7	0	0.95	0	0	5.67	0				1
At least 12 weeks less than 60 minutes without high RoB	Mean Difference	Random	-0.04	-0.29	0.21	29.3	0	1	0	0	0.31	0.75				0
Less than 12 weeks, less than 60 minutes without high RoB	Mean Difference	Random	0	0	0	0	0	0	0	0	0	0				0
Triglycerides Non Asian	Mean Difference	Random	-0.3	-0.83	0.24	100	26.45	0	92.44	0.19	1.1	0.27	26.45	0	96.22	2
At least 3 times/week	Mean Difference	Random	-0.05	-0.19	0.09	62.44	0	1	0	0	0.72	0.47				1
2 times/week	Mean Difference	Random	-0.71	-0.92	-0.5	37.56	0	1	0	0	6.56	0				0
Blood pressure																
SBP	Mean Difference	Random	-4.54	-6.41	-2.66	100	93.21	0	83.91	8.98	4.74	0	34.67	0	97.12	15
Asian	Mean Difference	Random	-5.52	-7.24	-3.8	81.41	50.52	0	80.2	5.6	6.28	0				10
Non-asian	Mean Difference	Random	1.79	0.07	3.51	18.59	3.66	0.45	0	0	2.04	0.04				4
SBP Asian, without high RoB	Mean Difference	Random	-7.1	-11.48	-2.71	100	16.82	0	82.17	14.86	3.17	0	11.46	0	91.27	3
at least 12 week at least 60 minutes without high RoB	Mean Difference	Random	-8.96	-11.73	-6.2	70.73	2.97	0.23	32.77	2	6.35	0				2
at least 12 weeks less than 60 minutes without high RoB	Mean Difference	Random	-2	-4.93	0.93	29.27	0	1	0	0	1.34	0.18				0
less than 12 weeks, more than 60 minutes without high RoB	Mean Difference	Random	0	0	0	0	0	0	0	0	0	0				0
less than 12 weeks, less than 60 minutes without high RoB	Mean Difference	Random	0	0	0	0	0	0	0	0	0	0				0
SBP Non Asian, without high RoB	Mean Difference	Random	1.79	0.07	3.51	100	3.66	0.45	0	0	2.04	0.04	0.01	0.92	0	4
at least 3 times/week without high RoB	Mean Difference	Random	0.18	-6.38	6.75	6.85	0.21	0.64	0	0	0.05	0.96				1
less than 2 times/week without high RoB	Mean Difference	Random	-0.3	-6.28	5.68	93.15	3.2	0.2	37.49	13.14	0.1	0.92				2
DBP	Mean Difference	Random	-2.23	-3.19	-1.27	100	45.21	0	69.03	1.58	4.57	0	8.93	0	88.8	14
Asian	Mean Difference	Random	-2.81	-3.86	-1.76	80.72	36.94	0	75.64	1.53	5.22	0				9
Non-asian	Mean Difference	Random	0.37	-1.43	2.17	19.28	2.27	0.69	0	0	0.41	0.69				4
DBP Asian, without high RoB	Mean Difference	Random	-6.16	-12.29	-0.02	100	21.14	0	90.54	26.36	1.97	0.05	20.96	0	95.23	2
At least 12 weeks at least 60 minutes without high RoB	Mean Difference	Random	-9.09	-11.88	-6.3	64.31	0.18	0.67	0	0	6.39	0				1
at least 12 weeks less than 60 minutes without high RoB	Mean Difference	Random	-1	-3.05	1.05	35.69	0	1	0	0	0.96	0.34				0
Less than 12 weeks without high RoB	Mean Difference	Random	0	0	0	0	0	0	0	0	0	0				0
Less than 12 weeks, less than 60 minutes without high RoB	Mean Difference	Random	0	0	0	0	0	0	0	0	0	0				0

DBP Non-Asian without high RoB	Mean Difference	Random	0.37	-1.43	2.17	100	2.27	0.69	0	0	0.41	0.69	0	0.98	0	4
at least 3 times/week without high RoB	Mean Difference	Random	0.29	-3.68	4.26	20.54	0.02	0.88	0	0	0.14	0.89				1
less than 2 times/week without high RoB	Mean Difference	Random	0.34	-1.84	2.51	79.46	2.25	0.32	11.13	0.44	0.3	0.76				2
Antioxidant																
Glutathione	Std. Mean Difference	Random	5.89	2.09	9.7	100	110.96	0	98.2	10.28	3.04	0	0.92	0.34	0	2
At least 12 weeks, at least 60 minutes	Std. Mean Difference	Random	10.22	-9.33	29.76	63.54	109.78	0	99.09	197.12	1.02	0.31				1
At least 12 weeks, less than 60 minutes	Std. Mean Difference	Random	0.62	0.15	1.09	36.46	0	1	0	0	2.61	0.01				0
Vitamin C	Std. Mean Difference	Random	19.25	-17.99	56.48	100	115.67	0	99.14	715.77	1.01	0.31	0	1	0	1
Vitamin E	Std. Mean Difference	Random	3.67	-3.01	10.35	100	74.68	0	98.66	22.93	1.08	0.28	0	1	0	1
SOD	Std. Mean Difference	Random	0.11	-1.03	1.24	100	40.49	0	92.59	1.23	0.18	0.86	1.45	0.49	0	3
At least 12 weeks, at least 60 minutes	Std. Mean Difference	Random	-0.49	-3.95	2.97	48.74	39.24	0	97.45	6.07	0.28	0.78				1
At least 12 weeks, less than 60 minutes	Std. Mean Difference	Random	0.38	-0.08	0.84	26.24	0	1	0	0	1.61	0.11				0
Less than 12 weeks, at least 60 minutes	Std. Mean Difference	Random	0.82	0.15	1.49	25.01	0	1	0	0	2.38	0.02				0
Inflammation																
TNF Alpha	Mean Difference	Random	-1.46	-1.93	-0.99	100	2.87	0.24	30.3	0.07	6.08	0	2.78	0.1	64.04	2
At least 12 weeks, less than 60 minutes	Mean Difference	Random	-1.25	-1.49	-1.01	68.62	0	1	0	0	10.33	0				0
Less than 12 weeks, less than 60 minutes	Mean Difference	Random	-1.92	-2.68	-1.17	31.38	0.09	0.77	0	0	4.99	0				1
IL-1	Mean Difference	Random	-0.48	-0.74	-0.21	100	1.77	0.18	43.35	0.02	3.53	0	0	1	0	1
IL-6	Mean Difference	Random	-0.62	-0.86	-0.37	100	0.23	0.63	0	0	4.95	0	0	1	0	1
HsCRP	Std. Mean Difference	Random	-0.38	-0.68	-0.07	100	4.82	0.19	37.8	0.04	2.44	0.01	4.41	0.11	54.61	3
Asian more than 12 weeks	Std. Mean Difference	Random	-0.06	-0.65	0.53	19.07	0	0	100	0	0.2	0.84				0
Asian less than 12 weeks	Std. Mean Difference	Random	-0.6	-0.91	-0.3	56.9	0.42	0.52	0	0	3.88	0				1
Non-Asian	Std. Mean Difference	Random	-0.1	-0.59	0.4	24.03	0	1	0	0	0.39	0.7				0
Anti-inflammation																
IL-10	Mean Difference	Random	0.39	0.15	0.63	100	2.58	0.28	22.5	0.01	3.16	0	0.58	0.45	0	2
At least 12 weeks less than 60 minutes	Mean Difference	Random	0.31	0.06	0.56	56.17	0	1	0	0	2.48	0.01				0
Less than 12 weeks, less than 60 minutes	Mean Difference	Random	0.52	0.04	0.99	43.83	1.91	0.17	47.7	0.06	2.14	0.03				1
