# Supplementary material for: Impact of yoga on cardiometabolic health in adults with overweight or obesity: A systematic review and meta-analysis of randomized controlled trials
Source: PLOS Glob Public Health. 2026 Apr 22;6(4):e0006174. doi: 10.1371/journal.pgph.0006174 (PMC13102220; doi:10.1371/journal.pgph.0006174)
Supplement: S6 Table — (DOCX) [file pgph.0006174.s008.docx]

GRADE analyses

| **Certainty assessment** | | | | | | | **№ of patients** | | **Effect** | | **Certainty** | **Importance** |
| --- | --- | --- | --- | --- | --- | --- | --- | --- | --- | --- | --- | --- |
| **№ of studies** | **Study design** | **Risk of bias** | **Inconsistency** | **Indirectness** | **Imprecision** | **Other considerations** | **Yoga** | **placebo** | **Relative (95% CI)** | **Absolute (95% CI)** |  |  |
| **Fasting Blood Glucose** | | | | | | | | | | | | |
| 10 | randomised trials | serious | not serious^a,b^ | not serious | not serious | none | 704 | 696 | - | MD **0.11 lower** (0.26 lower to 0.03 higher) | ⨁⨁⨁◯ Moderate^a,b^ |  |
| **Fasting Blood Glucose - Asia** | | | | | | | | | | | | |
| 8 | randomised trials | serious | not serious^a^ | not serious | not serious | none | 678 | 667 | - | MD **0.15 lower** (0.32 lower to 0.02 higher) | ⨁⨁⨁◯ Moderate^a^ |  |
| **Fasting Blood Glucose - Non Asia** | | | | | | | | | | | | |
| 2 | randomised trials | not serious | serious^c^ | not serious | serious^d^ | none | 26 | 29 | - | MD **0.03 higher** (0.25 lower to 0.3 higher) | ⨁⨁◯◯ Low^c,d^ |  |
| **Fasting Blood Glucose Asian without high RoB - at least 12 weeks at least 60 minutes** | | | | | | | | | | | | |
| 3 | randomised trials | not serious | not serious | not serious | serious^d^ | none | 139 | 138 | - | MD **0.01 SD lower** (0.08 lower to 0.06 higher) | ⨁⨁⨁◯ Moderate^d^ |  |
| **Fasting Blood Glucose Asian without high RoB - At least 12 weeks at least 60 minutes high FBG** | | | | | | | | | | | | |
| 1 | randomised trials | not serious | serious^e^ | not serious | serious^d,f^ | none | 8 | 8 | - | MD **0.68 SD lower** (0.88 lower to 0.48 lower) | ⨁⨁◯◯ Low^d,e,f^ |  |
| **Fasting Blood Glucose Asian without high RoB - Less than 12 weeks** | | | | | | | | | | | | |
| 1 | randomised trials | not serious | serious^e^ | not serious | serious^d^ | none | 28 | 29 | - | MD **0.32 SD higher** (0.17 higher to 0.47 higher) | ⨁⨁◯◯ Low^d,e^ |  |
| **Fasting Blood Glucose Asian without high RoB - At least 12 weeks less than 60 minutes** | | | | | | | | | | | | |
| 2 | randomised trials | not serious | not serious | not serious | serious^d^ | none | 112 | 109 | - | MD **0.68 SD lower** (1.8 lower to 0.45 higher) | ⨁⨁⨁◯ Moderate^d^ |  |
| **PPBG** | | | | | | | | | | | | |
| 4 | randomised trials | serious | not serious^b^ | not serious | serious^f^ | none | 524 | 520 | - | MD **0.2 SD lower** (0.62 lower to 0.23 higher) | ⨁⨁◯◯ Low^b,f^ |  |
| **PPBG - Asia** | | | | | | | | | | | | |
| 4 | randomised trials | serious | not serious^b^ | not serious | serious^f^ | none | 524 | 520 | - | MD **0.2 SD lower** (0.62 lower to 0.23 higher) | ⨁⨁◯◯ Low^b,f^ |  |
| **PPBG Asia without high RoB - Asian at least 12 week at least 60 minutes** | | | | | | | | | | | | |
| 2 | randomised trials | not serious | serious^c^ | not serious | serious^d,f^ | none | 105 | 108 | - | MD **0.02 SD higher** (0.59 lower to 0.63 higher) | ⨁⨁◯◯ Low^c,d,f^ |  |
| **PPBG Asia without high RoB - Asian less than 12 weeks** | | | | | | | | | | | | |
| 1 | randomised trials | not serious | serious^e^ | not serious | serious^d,f^ | none | 28 | 29 | - | SMD **0.45 SD lower** (0.68 lower to 0.22 lower) | ⨁⨁◯◯ Low^d,e,f^ |  |
| **HBA1c** | | | | | | | | | | | | |
| 4 | randomised trials | serious | serious^c^ | not serious | serious^f^ | none | 554 | 557 | - | MD **0.04 lower** (0.07lower to 0.02 lower) | ⨁◯◯◯ Very low^c,f^ |  |
| **HBA1c Asia without High RoB - At least 12 weeks, at least 60 minutes** | | | | | | | | | | | | |
| 2 | randomised trials | not serious | serious^c^ | not serious | serious^d,f^ | none | 105 | 108 | - | MD **0.05 SD lower** (0.13 lower to 0.02 higher) | ⨁⨁◯◯ Low^c,d,f^ |  |
| **HOMA-IR** | | | | | | | | | | | | |
| 3 | randomised trials | serious | serious^c^ | not serious | not serious | none | 480 | 472 | - | MD **0.87 lower** (1.64 lower to 0.1 lower) | ⨁⨁◯◯ Low^c^ |  |
| **HOMA-IR Asia Without High RoB - Less than 12 weeks** | | | | | | | | | | | | |
| 1 | randomised trials | not serious | serious^e^ | not serious | serious^d^ | none | 28 | 29 | - | MD **0.5 SD lower** (0.7 lower to 0.3 lower) | ⨁⨁◯◯ Low^d,e^ |  |
| **HOMA-IR Asia Without High RoB - At least 12 weeks** | | | | | | | | | | | | |
| 1 | randomised trials | not serious | serious^e^ | not serious | serious^d^ | none | 61 | 60 | - | MD **2.64 SD lower** (3.54 lower to 1.74 lower) | ⨁⨁◯◯ Low^d,e^ |  |

**CI:** confidence interval; **MD:** mean difference; **SMD:** standardised mean difference

#### Explanations

a. Heterogeneities were explained by the dose-response effect

b. Heterogeneities were explained by the ethnic-differences

c. Unexplained heterogeneity

d. Small sample size

e. Single study

f. Effect size crossed the MCID

| **Certainty assessment** | | | | | | | **№ of patients** | | **Effect** | | **Certainty** | **Importance** |
| --- | --- | --- | --- | --- | --- | --- | --- | --- | --- | --- | --- | --- |
| **№ of studies** | **Study design** | **Risk of bias** | **Inconsistency** | **Indirectness** | **Imprecision** | **Other considerations** | **Yoga** | **placebo** | **Relative (95% CI)** | **Absolute (95% CI)** |  |  |
| **VLDL** | | | | | | | | | | | | |
| 2 | randomised trials | serious | not serious | not serious | serious^a^ | none | 133 | 139 | - | MD **0.04 lower** (0.08 lower to 0 ) | ⨁⨁◯◯ Low^a^ |  |
| **LDL** | | | | | | | | | | | | |
| 9 | randomised trials | serious | not serious^b,c^ | not serious | not serious | none | 675 | 682 | - | MD **0.04 lower** (0.09 lower to 0 ) | ⨁⨁⨁◯ Moderate^b,c^ |  |
| **LDL - Asian** | | | | | | | | | | | | |
| 6 | randomised trials | serious | not serious^c^ | not serious | not serious | none | 620 | 619 | - | MD **0.07 lower** (0.12 lower to 0.02 lower) | ⨁⨁⨁◯ Moderate^c^ |  |
| **LDL - Non-asian** | | | | | | | | | | | | |
| 3 | randomised trials | not serious | not serious | not serious | serious^a^ | none | 55 | 63 | - | MD **0.09 higher** (0.02 lower to 0.2 higher) | ⨁⨁⨁◯ Moderate^a^ |  |
| **LDL Asian without High RoB - At least 12 weeks at least 60 minutes** | | | | | | | | | | | | |
| 2 | randomised trials | not serious | not serious | not serious | serious^a^ | none | 99 | 99 | - | MD **0.48 lower** (0.62 lower to 0.33 lower) | ⨁⨁⨁◯ Moderate^a^ |  |
| **LDL Asian without High RoB - At least 12 weeks less than 60 minutes** | | | | | | | | | | | | |
| 1 | randomised trials | not serious | serious^d^ | not serious | serious^d^ | none | 51 | 49 | - | MD **0.07 lower** (0.19 lower to 0.05 higher) | ⨁⨁◯◯ Low^d^ |  |
| **LDL Non Asian** | | | | | | | | | | | | |
| 3 | randomised trials | not serious | not serious | not serious | serious^a^ | none | 55 | 63 | - | MD **0.09 higher** (0.02 lower to 0.2 higher) | ⨁⨁⨁◯ Moderate^a^ |  |
| **HDL** | | | | | | | | | | | | |
| 10 | randomised trials | serious | not serious^b,c^ | not serious | not serious | none | 735 | 743 | - | MD **0.06 higher** (0.01 higher to 0.10 higher) | ⨁⨁⨁◯ Moderate^b,c^ |  |
| **HDL - Asian** | | | | | | | | | | | | |
| 6 | randomised trials | serious | not serious^b^ | not serious | not serious | none | 622 | 614 | - | MD **0.08 higher** (0 to 0.15 higher) | ⨁⨁⨁◯ Moderate^b^ |  |
| **HDL - Non-asian** | | | | | | | | | | | | |
| 4 | randomised trials | not serious | not serious | not serious | serious^a^ | none | 113 | 129 | - | MD **0.03 higher** (0.01 lower to 0.06 higher) | ⨁⨁⨁◯ Moderate^a^ |  |
| **HDL Asian without high RoB - At least 12 weeks at least 60 minutes** | | | | | | | | | | | | |
| 2 | randomised trials | not serious | serious^e^ | not serious | serious^a^ | none | 101 | 99 | - | MD **0.12 higher** (0.03 higher to 0.2 higher) | ⨁⨁◯◯ Low^a,e^ |  |
| **HDL Asian without high RoB - At least 12 weeks, less than 60 minutes** | | | | | | | | | | | | |
| 1 | randomised trials | not serious | serious^d^ | not serious | serious^a^ | none | 51 | 49 | - | MD **0.04 higher** (0.01 lower to 0.08 higher) | ⨁⨁◯◯ Low^a,d^ |  |
| **Total cholesterol** | | | | | | | | | | | | |
| 9 | randomised trials | serious | not serious^c^ | not serious | not serious | none | 696 | 706 | - | MD **0.05 lower** (0.14 lower to 0.03 higher) | ⨁⨁⨁◯ Moderate^c^ |  |
| **Total cholesterol - Asian** | | | | | | | | | | | | |
| 5 | randomised trials | serious | not serious^b^ | not serious | not serious | none | 583 | 577 | - | MD **0.1 lower** (0.22 lower to 0.02 higher) | ⨁⨁⨁◯ Moderate^b^ |  |
| **Total cholesterol - Non-asian** | | | | | | | | | | | | |
| 4 | randomised trials | not serious | not serious | not serious | serious^a^ | none | 113 | 129 | - | MD **0.01 higher** (0.07 lower to 0.10 higher) | ⨁⨁⨁◯ Moderate^a^ |  |
| **Total cholesterol Asian without high RoB - At least 12 weeks at least 60 minutes** | | | | | | | | | | | | |
| 2 | randomised trials | not serious | serious^e^ | not serious | serious^a^ | none | 99 | 99 | - | MD **0.31 lower** (0.94 lower to 0.31 higher) | ⨁⨁◯◯ Low^a,e^ |  |
| **Total cholesterol Asian without high RoB - At least 12 weeks less than 60 minutes** | | | | | | | | | | | | |
| 1 | randomised trials | not serious | serious^d^ | not serious | serious^a^ | none | 51 | 49 | - | MD **0.05 lower** (0.19 lower to 0.09 higher) | ⨁⨁◯◯ Low^a,d^ |  |
| **Triglycerides** | | | | | | | | | | | | |
| 9 | randomised trials | serious | not serious^b^ | not serious | not serious | none | 675 | 679 | - | MD **0.26 lower** (0.42 lower to 0.11 lower) | ⨁⨁⨁◯ Moderate^b^ |  |
| **Triglycerides - Asian** | | | | | | | | | | | | |
| 6 | randomised trials | serious | not serious^b^ | not serious | not serious | none | 620 | 616 | - | MD **0.23 lower** (0.4 lower to 0.07 lower) | ⨁⨁⨁◯ Moderate^b^ |  |
| **Triglycerides - Non-asian** | | | | | | | | | | | | |
| 3 | randomised trials | not serious | not serious^b^ | not serious | serious^a^ | none | 55 | 63 | - | MD **0.3 lower** (0.83 lower to 0.24 higher) | ⨁⨁⨁◯ Moderate^a,b^ |  |
| **Triglycerides Asian without high RoB - At least 12 weeks at least 60 minutes** | | | | | | | | | | | | |
| 2 | randomised trials | not serious | not serious | not serious | serious^a^ | none | 99 | 101 | - | MD **0.35 lower** (0.47 lower to 0.23 lower) | ⨁⨁⨁◯ Moderate^a^ |  |
| **Triglycerides Asian without high RoB - At least 12 weeks less than 60 minutes** | | | | | | | | | | | | |
| 1 | randomised trials | not serious | serious^d^ | not serious | serious^a^ | none | 51 | 49 | - | MD **0.04 lower** (0.29 lower to 0.21 higher) | ⨁⨁◯◯ Low^a,d^ |  |

**CI:** confidence interval; **MD:** mean difference

#### Explanations

a. Small sample size

b. Heterogeneities were explained by the dose-response effect

c. Heterogeneities were explained by the ethnic-differences

d. Singe study

e. Unexplained heterogeneity

| **Certainty assessment** | | | | | | | **№ of patients** | | **Effect** | | **Certainty** | **Importance** |
| --- | --- | --- | --- | --- | --- | --- | --- | --- | --- | --- | --- | --- |
| **№ of studies** | **Study design** | **Risk of bias** | **Inconsistency** | **Indirectness** | **Imprecision** | **Other considerations** | **Yoga** | **placebo** | **Relative (95% CI)** | **Absolute (95% CI)** |  |  |
| **SBP** | | | | | | | | | | | | |
| 17 | randomised trials | serious | not serious^a,b^ | not serious | not serious | none | 552 | 534 | - | MD **4.35 lower** (6.10 lower to 2.60 lower) | ⨁⨁⨁◯ Moderate^a,b^ |  |
| **SBP - Asian** | | | | | | | | | | | | |
| 11 | randomised trials | serious | not serious^a^ | not serious | not serious | none | 383 | 367 | - | MD **5.52 lower** (7.24 lower to 3.8 lower) | ⨁⨁⨁◯ Moderate^a^ |  |
| **SBP - Non-asian** | | | | | | | | | | | | |
| 6 | randomised trials | serious | not serious | not serious | not serious^c^ | none | 169 | 167 | - | MD 0.39 **lower** (3.41 lower to 2.64 higher) | ⨁⨁⨁◯ Moderate^c^ |  |
| **SBP Asian, without high RoB - at least 12 week at least 60 minutes** | | | | | | | | | | | | |
| 3 | randomised trials | not serious | serious^d^ | not serious | serious^c^ | none | 50 | 51 | - | MD **8.96 lower** (11.73 lower to 6.2 lower) | ⨁⨁◯◯ Low^c,d^ |  |
| **SBP Asian, without high RoB - at least 12 weeks less than 60 minutes** | | | | | | | | | | | | |
| 1 | randomised trials | not serious | serious^e^ | not serious | serious^c^ | none | 51 | 49 | - | MD **2 lower** (4.93 lower to 0.93 higher) | ⨁⨁◯◯ Low^c,e^ |  |
| **DBP** | | | | | | | | | | | | |
| 16 | randomised trials | serious | not serious^a,b^ | not serious | serious^f^ | none | 524 | 506 | - | MD **2.06 lower** (2.97 lower to 1.15 lower) | ⨁⨁◯◯ Low^a,b,f^ |  |
| **DBP - Asian** | | | | | | | | | | | | |
| 10 | randomised trials | serious | not serious^a^ | not serious | serious^f^ | none | 355 | 339 | - | MD **2.81 lower** (3.86 lower to 1.76 lower) | ⨁⨁◯◯ Low^a,f^ |  |
| **DBP - Non-asian** | | | | | | | | | | | | |
| 6 | randomised trials | serious | not serious | not serious | not serious^c^ | none | 169 | 167 | - | MD **0.00 lower** (1.26 lower to 1.26 higher) | ⨁⨁⨁◯ Moderate^c^ |  |
| **DBP Asian, without high RoB - At least 12 weeks at least 60 minutes** | | | | | | | | | | | | |
| 2 | randomised trials | not serious | not serious | not serious | serious^c^ |  | 22 | 23 | - | MD **9.09 lower** (11.88 lower to 6.3 lower) | -^c^ |  |
| **DBP Asian, without high RoB - at least 12 weeks less than 60 minutes** | | | | | | | | | | | | |
| 1 | randomised trials | not serious | serious^e^ | not serious | serious^c^ |  | 51 | 49 | - | MD **1 lower** (3.05 lower to 1.05 higher) | -^c,e^ |  |

**CI:** confidence interval; **MD:** mean difference

#### Explanations

a. Heterogeneity was explained by dose-response effects

b. Heterogeneity was explained by ethnic differences

c. Small sample size

d. Unexplained heterogeneity

e. Single study

f. Effect size crossed MCID

| **Certainty assessment** | | | | | | | **№ of patients** | | **Effect** | | **Certainty** | **Importance** |
| --- | --- | --- | --- | --- | --- | --- | --- | --- | --- | --- | --- | --- |
| **№ of studies** | **Study design** | **Risk of bias** | **Inconsistency** | **Indirectness** | **Imprecision** | **Other considerations** | **Yoga** | **placebo** | **Relative (95% CI)** | **Absolute (95% CI)** |  |  |
| **Glutathione** | | | | | | | | | | | | |
| 3 | randomised trials | serious | serious^a^ | not serious | serious^b^ | none | 85 | 82 | - | SMD **5.89 higher** (2.09 higher to 9.7 higher) | ⨁◯◯◯ Very low^a,b^ |  |
| **Glutathione - At least 12 weeks, at least 60 minutes** | | | | | | | | | | | | |
| 2 | randomised trials | not serious | serious^a^ | not serious | serious^b^ | none | 48 | 45 | - | SMD **10.22 higher** (9.33 lower to 29.76 higher) | ⨁⨁◯◯ Low^a,b^ |  |
| **Glutathione - At least 12 weeks, less than 60 minutes** | | | | | | | | | | | | |
| 1 | randomised trials | serious | serious^c^ | not serious | serious^b^ | none | 37 | 37 | - | SMD **0.62 higher** (0.15 higher to 1.09 higher) | ⨁◯◯◯ Very low^b,c^ |  |
| **Vitamin C** | | | | | | | | | | | | |
| 2 | randomised trials | not serious | serious^a^ | not serious | serious^b^ | none | 48 | 45 | - | MD **56.52 higher** (49.71 lower to 162.75 higher) | ⨁⨁◯◯ Low^a,b^ |  |
| **Vitamin E** | | | | | | | | | | | | |
| 2 | randomised trials | not serious | serious^a^ | not serious | serious^b^ | none | 48 | 45 | - | MD **9.75 higher** (3.77 lower to 23.28 higher) | ⨁⨁◯◯ Low^a,b^ |  |
| **SOD** | | | | | | | | | | | | |
| 4 | randomised trials | serious | serious^a^ | not serious | serious^b^ | none | 103 | 101 | - | SMD **0.11 higher** (1.03 lower to 1.24 higher) | ⨁◯◯◯ Very low^a,b^ |  |

**CI:** confidence interval; **MD:** mean difference; **SMD:** standardised mean difference

#### Explanations

a. Unexplained heterogeneity

b. Small sample size

c. Single study

| **Certainty assessment** | | | | | | | | | | **№ of patients** | | **Effect** | | | **Certainty** | | **Importance** | |  |  |
| --- | --- | --- | --- | --- | --- | --- | --- | --- | --- | --- | --- | --- | --- | --- | --- | --- | --- | --- | --- | --- |
| **№ of studies** | | **Study design** | | **Risk of bias** | | **Inconsistency** | **Indirectness** | **Imprecision** | **Other considerations** | **Yoga** | **placebo** | **Relative (95% CI)** | **Absolute (95% CI)** | |  |  |  |  |  |  |
| **TNF Alpha** | | | | | | | | | | | | | | | | | | |  |  |
| 3 | | randomised trials | | serious | | serious^a^ | not serious | serious^b^ | none | 119 | 129 | - | MD **1.46 lower** (1.93 lower to 0.99 lower) | | ⨁◯◯◯ Very low^a,b^ | |  | |  |  |
| **IL-1** | | | | | | | | | | | | | | | | | | |  |  |
| 2 | | randomised trials | | serious | | serious^a^ | not serious | serious^b^ | none | 82 | 92 | - | MD **0.48 lower** (0.74 lower to 0.21 lower) | | ⨁◯◯◯ Very low^a,b^ | |  | |  |  |
| **IL-6** | | | | | | | | | | | | | | | | | | |  |  |
| 3 | | randomised trials | | serious | | not serious | not serious | serious^b^ | none | 140 | 158 | - | MD **0.63 lower** (0.87 lower to 0.39 lower) | | ⨁⨁◯◯ Low^b^ | |  | |  |  |
| **HsCRP** | | | | | | | | | | | | | | | | | | |  |  |
| 4 | | randomised trials | | serious | | serious^a^ | not serious | serious^b^ | none | 129 | 155 | - | SMD **0.38 lower** (0.68 lower to 0.07 lower) | | ⨁◯◯◯ Very low^a,b^ | |  | |  |  |
| **IL-10** | | | | | | | | | | | | | | | | | | | | |
| 3 | | randomised trials | | serious | | not serious^a^ | | not serious | serious^b^ | none | 119 | 129 | - | | MD **0.39 higher** (0.15 higher to 0.63 higher) | | ⨁⨁◯◯ Low^a,b^ | |  | |

**CI:** confidence interval; **MD:** mean difference

**CI:** confidence interval; **MD:** mean difference; **SMD:** standardised mean difference

#### Explanations

a. Unexplained heterogeneity

b. Small sample size
