# Supplementary material for: Impact of yoga on cardiometabolic health in adults with overweight or obesity: A systematic review and meta-analysis of randomized controlled trials
Source: PLOS Glob Public Health. 2026 Apr 22;6(4):e0006174. doi: 10.1371/journal.pgph.0006174 (PMC13102220; doi:10.1371/journal.pgph.0006174)
Supplement: Attachment — Submitted filename: Responses to reviewers.docx [file pgph.0006174.s011.docx]

Dear editor and reviewers,

We would like to thank the Editor and the reviewers for their thoughtful, constructive, and insightful comments, which have substantially improved the clarity, transparency, and clinical relevance of this manuscript. In response, we have updated the literature search to ensure currency, incorporated newly eligible studies, and revised the analyses and results accordingly. We strengthened the Abstract by adding key quantitative findings and clarifying study inclusion criteria,and refined the Introduction to better distinguish the unique contribution of this review. The Methods section was clarified to improve transparency around eligibility decisions, handling of combined interventions, assessment of publication bias, verification of data extracted from graphs, and comparator rationale. We reduced repetition in the Discussion, expanded the consideration of generalizability and key limitations (including comorbidities and dose–response analysis), and more prominently emphasized the clinical interpretation of findings, particularly in relation to minimal clinically important differences. Figures and supplementary materials were also revised to meet journal requirements. We believe these revisions have meaningfully strengthened the manuscript and addressed all comments raised.

Best regards,

Dr. Alexios Batrakoulis

| Editor | Response | Evidence |
| --- | --- | --- |
| 1.  Please provide separate figure files in .tif or .eps format.  For more information about figure files please see our guidelines:  <https://journals.plos.org/globalpublichealth/s/figures>  <https://journals.plos.org/globalpublichealth/s/figures#loc-file-requirements> | Thank you for reminding us about the journal’s requirements. Separate figure files have now been provided in EPS format | Separate figure files in eps format have been provided |
| 2. We note that you have included your Figures within the body of your manuscript. Please remove the Figures from the body of your manuscript and upload them as separate Figure files. | Thank you for noting this. We have now removed the figures from the body of the manuscript and uploaded them as separate figure files. | Figures have been removed from the body of manuscript |
| 3. As required by our policy on Data Availability, please ensure your manuscript or supplementary information includes the following:  A numbered table of all studies identified in the literature search, including those that were excluded from the analyses.  For every excluded study, the table should list the reason(s) for exclusion.  If any of the included studies are unpublished, include a link (URL) to the primary source or detailed information about how the content can be accessed.  A table of all data extracted from the primary research sources for the systematic review and/or meta-analysis. The table must include the following information for each study:  Name of data extractors and date of data extraction  Confirmation that the study was eligible to be included in the review. | Thank you for the clarification regarding the Data Availability requirements. We confirm that all requested information has been provided in the supplementary materials. | All included studies, including those that were excluded from the meta-analyses have been reported in the S4 table  All excluded studies with reason for exclusion have been reported in the S4 table  All data extracted from the primary research sources for the meta-analyses have been reported in the S2 file by including the name of data extractors |
| 4. In the online submission form, you indicated that “The data that support the findings of this study are available from the corresponding author upon reasonable request.”.  All PLOS journals now require all data underlying the findings described in their manuscript to be freely available to other researchers, either  1. In a public repository,  2. Within the manuscript itself, or  3. Uploaded as supplementary information. | Thank you for this clarification. We have revised the Data Availability statement and ensured that all data underlying the findings are freely available via the manuscript and supplementary information. | We have uploaded all data that support the findings in the manuscript and supplementary files |
| Reviewer 1 |  |  |
| 1.) Abstract The concluding statement in the abstract may be strengthened by explicitly stating some key numerical findings of the review. | Thank you for this helpful suggestion. We have revised the concluding statement of the abstract to include key numerical findings from the meta-analyses, including the pooled effect estimates for main outcomes, to make the take-home message more explicit and informative. | Twenty-two randomized controlled trials involving 2,189 participants were included in the meta-analyses, which demonstrated that yoga practices likely have substantial beneficial effects on systolic blood pressure (-4.35 mmHg), diastolic blood pressure (-2.06 mmHg) and modest effects below the minimal clinically important differences on lipid profiles (low-density lipoprotein cholesterol -0.08 mmol/L, high-density lipoprotein cholesterol +0.06 mmol/L) with moderate quality evidence. (lines 62-66) |
| 2.) Introduction The gap in BMI-specific analysis in previous reviews is properly identified; however, the unique contributions of this review could be highlighted more by more clearly distinguishing it from earlier meta-analyses conducted. | Thank you for this helpful suggestion. We have revised the Introduction to more clearly distinguish the unique contribution of our review from earlier meta-analyses by explicitly highlighting our focus on BMI-defined populations and our synthesis of a broader set of cardiometabolic outcomes | Therefore, this systematic review and meta-analysis aimed to assess the impact of yoga practice on a wide spectrum of cardiometabolic health outcomes in individuals with overweight or obesity, focusing specifically on BMI-defined populations and synthesizing a broader set of outcomes than prior reviews. (lines 121-124) |
| 3.) Materials and Methods Clarify if publication bias was statistically assessed in addition to funnel plots. Also add brief explanation on how “high risk of bias” studies were defined. | Thank you for this helpful comment. We have clarified in the Methods that publication bias was assessed both visually (via funnel plots) and statistically using Egger’s test. We have also added a brief definition of how studies were classified as having a high risk of bias to improve transparency and reproducibility | Funnel plots were generated to assess publication bias through visual inspection of funnel plot symmetry, and this was supplemented by Egger’s test. (lines 244 to 246)  We assessed a study as having a high risk of bias when there is a high risk of bias in at least one domain. (lines 225 to 226) |
| 4.) Results The results section is detailed and clearly presented with subgroup and sensitivity analyses. The statistical analysis is done appropriately. | Thank you for your positive feedback. We are grateful that you found the Results section clear and appropriately analysed, including the subgroup and sensitivity analyses. |  |
| 5.) Discussion The discussion has some repetition of results. Consider reducing. | Thank you for this helpful suggestion. We have reduced repetition in the Discussion by removing repeated numerical effect estimates (particularly for HOMA-IR, PPBG, and HbA1c), while retaining the fasting blood glucose (FBG) estimate to aid interpretation and comparison with previous studies. | Our findings indicated that yoga practice might result in small beneficial effects on both metabolism and clinical markers of glucose, as demonstrated by its effect on HOMA-IR, FBG (-0.11 mmol/L), PPBG and HbA1c in people with excess body weight |
| 6.) Conclusion The final paragraph of the conclusion section will benefit from explanation of the relevance of findings to their implications for public health policy and clinical guidelines. | Thank you for this helpful suggestion. We have revised the final paragraph of the Conclusion to more explicitly describe the relevance of our findings to public health policy and clinical guideline development, particularly in relation to lifestyle-based management of overweight and obesity | These findings support the use of yoga as an adjunctive treatment option for the clinical management of poor metabolic health with potential implications for future clinical guideline development and public health policy focused on lifestyle-based management of overweight and obesity. |
| 7.) Figures Please consider uploading the figures in a higher resolution for better clarity. | Thank you for this suggestion. We have now uploaded high-resolution versions of all figures as separate files in EPS format to improve clarity. | High resolution figures in an eps format have been uploaded as separate files |
| Reviewer 2 |  |  |
| 1. Line 54: The search cutoff date is November 2024, indicating that over a year has passed since the searches were performed. Both the Cochrane Collaboration and the Joanna Briggs Institute advise updating literature searches after 12 months, as new relevant studies meeting inclusion criteria may have been published. I recommend updating the search to find and include any recent articles that are relevant. | Thank you for this important suggestion. This has improved the quality and timeliness of our review. We have updated the searches, incorporated newly eligible studies, and updated the analyses and manuscript accordingly. | Updated searches on 25^th^ December 2025 have been conducted and two additional eligible studies have been included in the analyses. All relevant texts, figures, and tables have been revised accordingly |
| 2. Please include some quantitative results from the meta-analysis in the abstract.t. | Thank you for this helpful suggestion. We have revised the abstract to include key quantitative results from the meta-analyses, including the pooled effect estimates for the main outcomes. | Twenty-two randomized controlled trials involving 2,189 participants were included in the meta-analyses, which demonstrated that yoga practices likely have substantial beneficial effects on systolic blood pressure (-4.35 mmHg), diastolic blood pressure (-2.06 mmHg) and modest effects below the minimal clinically important differences on lipid profiles (low-density lipoprotein cholesterol -0.08 mmol/L, high-density lipoprotein cholesterol +0.06 mmol/L) with moderate quality evidence. (lines 62-66) |
| 3. Lines 126-132: The use of WHO BMI thresholds (≥23 for Asians, ≥25 for others) is appropriate. However, the manuscript states that for studies not explicitly mentioning overweight/obesity, the baseline BMI was extracted from the full text. Please clarify the process if only the mean group BMI met the threshold, but individual participant data was not available. Was the study included if the mean BMI of the intervention/control group met the threshold, even if some participants individually did not? This has implications for the specificity of the findings to an overweight/obese population. | Thank you for this important clarification. We have revised the Methods to explicitly describe how eligibility was determined when studies did not clearly report overweight/obesity status. Specifically, where individual participant data were not available, eligibility was determined using the baseline BMI reported for the study sample, applying the WHO/WHO Asia-Pacific thresholds as specified. | We included the study if the participants were considered to have a high BMI according to the WHO and WHO Asia–Pacific guidelines [35, 36], with thresholds of more than or equal to 23 and 25 for Asian and other ethnicities, respectively. If the study did not explicitly mention overweight, high BMI, or obesity in the abstract or title, we found the participants' baseline BMI in the full text if individual participant data was not available.  (lines 133 to 136) |
| 4. Lines 126-132: The exclusion of participants with comorbidities (T2DM, CAD, etc.) is justified to isolate the effect of yoga. However, this significantly limits the clinical applicability of the findings, as overweight/obesity often coexists with these conditions. This should be highlighted as a key limitation affecting generalizability to the broader target population in clinical practice (see also Lines 564-566). | Thank you for this important point. We have revised the Discussion to more explicitly acknowledge that excluding participants with comorbidities may improve internal validity (by reducing confounding) but limits the clinical applicability and generalisability of our findings, given the common co-occurrence of comorbidities among individuals with overweight or obesity | Third, the exclusion of participants with overweight/obesity having comorbidities from the present study may have resulted in a smaller total sample size. This decision helped to isolate prevention-oriented effects of yoga on cardiometabolic outcomes by reducing confounding from established obesity-related disease. However, it also reduces generalizability to clinical practice, given the high prevalence of comorbidities among individuals with overweight or obesity. On contrary, this decision impacted the generalizability of the current systematic review results to the broader target population in clinical practice as there is high prevalence of comorbidities among individuals with overweight or obesity. (lines 682 to 687) |
| 5. The definition of "yoga" as a complex intervention is clear. However, for the "combined intervention" category (yoga plus other health interventions), the protocol states the single or complementary effect of yoga must be examinable. Please specify in the methods how this was determined from the study design (e.g., only included if there was a yoga-only arm vs. a control arm, allowing isolation of yoga's effect). | Thank you for this helpful clarification. We have specified in the Methods how combined-intervention studies were handled and clarified that we only included studies where the effect of yoga could be examined either as a single effect or a complementary effect. Examples have been added to illustrate both scenarios. | We included studies that combined yoga with other health interventions if the single effect (yoga+diet vs yoga vs control) or the complementary effect of yoga could be examined (yoga+pharmacotherapy vs pharmacotherapy). (lines 146 to 148) |
| 6. The comparator includes "other types of physical activity interventions." Consider adding a brief rationale for this choice (e.g., to assess yoga's efficacy relative to other common exercise prescriptions). This strengthens the review's aim to inform exercise selection. | Thank you for this helpful suggestion. We have added a brief rationale in the Methods explaining why other types of physical activity interventions were included as comparators, to allow assessment of yoga’s efficacy relative to commonly prescribed exercise modalities and to strengthen the review’s relevance for informing physical activity/exercise type selection. | We included any studies that allowed us to compare the single effect or the complementary effect of yoga to inactive control or other types of physical activity interventions. By assessing yoga’s efficacy relative to other physical activity or exercise intervention, this decision could strengthen the current review to inform physical activity type selection. (lines 151 to 154) |
| 7. The vote-counting approach for narrative synthesis is clearly described. The use of WebPlotDigitizer for data extraction from graphs is noted, which is a common practice. Consider adding a sentence on how data extracted this way was verified (e.g., by a second reviewer) to ensure accuracy. | Thank you for this helpful suggestion. We have added a sentence in the Methods to clarify how data extracted using WebPlotDigitizer were verified, including independent extraction by two reviewers and reconciliation of any discrepancies to ensure accuracy  $ | We used the WebPlotDigitizer (Automeris LLC, USA) to extract the change from the baseline and its standard deviation (SD) from the graphical data [47]. Two reviewers extracted the values independently; where minor differences occurred, the values were re-checked against the original graphs and a final value was agreed,  (lines 243 to 246) |
| 8. The rationale for subgroup analyses (ethnicity, dose) is well-founded. However, the protocol mentions dose–response effects (Lines 256-260) but the primary subgroup analyses in the results (Lines 389-406) focus on categorical cut-offs (e.g., ≥12 weeks, ≥60 min/session). Consider if a meta-regression analyzing dose (total minutes of yoga) as a continuous variable was explored and, if not, mention it as a limitation for future research. This could provide a more nuanced dose-response relationship. | Thank you for this helpful suggestion. We agree that meta-regression could offer a more nuanced assessment of dose–response relationships, but the limited number of available studies did not allow us to conduct this analysis. We have clarified in the Limitation section that meta-regression was not conducted and noted this as a limitation | Finally, we were unable to conduct meta-regression to statistically assess the dose-response effects due to the limited number of available primary studies. (lines 694 to 696) |
| 9. In the "Quality of evidence" section (Lines 419-447), it is stated that for most outcomes the results did not surpass the MCID. This is a critical point for clinical interpretation. Consider emphasizing this more prominently in the Abstract (Lines 63-65) and Discussion to ensure readers appreciate that while statistically significant, changes in lipid profiles, for example, were modest in clinical magnitude. | Thank you for this important comment. We agree that highlighting whether effects exceed the minimal clinically important difference (MCID) is critical for clinical interpretation. We have therefore revised both the Abstract and the Discussion to more prominently emphasise that effects, particularly for lipid profile outcomes. | Twenty-two randomized controlled trials involving 2,189 participants were included in the meta-analyses, which demonstrated that yoga practices likely have substantial beneficial effects on systolic blood pressure (-4.35 mmHg), diastolic blood pressure (-2.06 mmHg) and modest effects below the minimal clinically important differences on lipid profiles (low-density lipoprotein cholesterol -0.08 mmol/L, high-density lipoprotein cholesterol +0.06 mmol/L) with moderate quality evidence.  (lines 62 to 66)  In our meta-analysis, yoga resulted in subclinical beneficial changes in VLDL, HDL, and TG but not in TC or LDL. Importantly, these improvements were observed only in Asians, suggesting that ethnic differences in lipid metabolism align with the findings of a previous systematic review [25]. (lines 616 to 618) |
| 10. Lines 60-61: The statement "While 25 studies did not specifically recruit individuals with obesity, the average BMI... fell within the overweight or obese range" is important but slightly ambiguous. Rephrase for clarity: e.g., "Although most studies (25/28) did not explicitly recruit an overweight/obese population, the mean baseline BMI of participants met our inclusion criteria for overweight/obesity." | Thank you for this helpful suggestion. We have rephrased this sentence in the Abstract for clarity, following your suggested wording | Although most studies (25/28) did not explicitly recruit individuals with obesity, the mean baseline BMI of participants met our inclusion criteria for overweight or obesity. |
